# Proteomics and SSH Analyses of ALA-Promoted Fruit Coloration and Evidence for the Involvement of a MADS-Box Gene, *MdMADS1*

**DOI:** 10.3389/fpls.2016.01615

**Published:** 2016-11-07

**Authors:** Xinxin Feng, Yuyan An, Jie Zheng, Miao Sun, Liangju Wang

**Affiliations:** College of Horticulture, Nanjing Agricultural UniversityNanjing, China

**Keywords:** anthocyanin, apple, 5-aminolevulinic acid (ALA), proteomics, suppression subtractive hybridization (SSH), *MdMADS1*

## Abstract

Skin color is a key quality attribute of fruits and how to improve fruit coloration has long been a major concern. 5-Aminolevulinic acid (ALA), a natural plant growth regulator, can significantly increase anthocyanin accumulation in fruit skin and therefore effectively improve coloration of many fruits, including apple. However, the molecular mechanism how ALA stimulates anthocyanin accumulation in fruit skin remains unknown. Here, we investigated the impact of ALA on apple skin at the protein and mRNA levels. A total of 85 differentially expressed proteins in apple skins between ALA and water treatment (control) were identified by complementary gel-based and gel-free separation techniques. Most of these differentially expressed proteins were up-regulated by ALA. Function analysis suggested that 87.06% of the ALA-responsive proteins were associated with fruit ripening. To further screen ALA-responsive regulators, we constructed a subtracted cDNA library (tester: ALA treatment; driver: control) and obtained 104 differentially expressed unigenes, of which 38 unigenes were indicators for the fruit ripening-related genes. The differentially changed proteins and transcripts did not correspond well at an individual level, but showed similar regulated direction in function at the pathway level. Among the identified fruit ripening-related genes, the expression of *MdMADS1*, a developmental transcription regulator of fruit ripening, was positively correlated with expression of anthocyanin biosynthetic genes (*MdCHS, MdDFR, MdLDOX, and MdUFGT*) in apple skin under ALA treatment. Moreover, overexpression of *MdMADS1* enhanced anthocyanin content in transformed apple calli, which was further enhanced by ALA. The anthocyanin content in *MdMADS1-*silenced calli was less than that in the control with ALA treatment, but higher than that without ALA treatment. These results indicated that *MdMADS1* is involved in ALA-induced anthocyanin accumulation. In addition, anthocyanin-related verification in apple calli suggested that the regulation of *MdMADS1* on anthocyanin biosynthesis was partially independent of fruit ripening process. Taken together, our findings provide insight into the mechanism how ALA regulates anthocyanin accumulation and add new information on transcriptase regulators of fruit coloration.

## Introduction

Skin color is a key quality attribute of apple fruit, and hence one of the most important factors determining apple market acceptance. Generally, well-colored red cultivars are preferred because consumers always associate the red color with some indication of fruit quality, such as maturity, nutrition, taste, and flavor. At commercial apple orchards of southern China, poor red coloration has been an important limiting factor of apple commodity value. Thus, how to promote apple fruit coloration has become a major concern.

Many attempts have been applied to improve red coloration in apple fruits. The traditional fruit production practices contain paper bagging (Ju, 1998) and covering the orchard floor with reflecting films (Meinhold et al., 2010). However, these methods demand a mass of manpower, material resources, and time, or even bring orchard pollution. By contrast, the application of plant growth substances has been proposed as an economically viable alternative. 5-Aminolevulinic acid (ALA), an essential biosynthetic precursor of tetrapyrrole molecules, acts as a new-type plant growth regulator. ALA has gained increasing attention because of its multiple physiological roles in plants, such as increasing plant resistance to various stresses, and improving plant photosynthesis (Akram and Ashraf, 2013; Murooka and Tanaka, 2014). In fruit production, ALA has been demonstrated to be effective for the promotion of fruit coloration in several fruit crops, including apple (Xie et al., 2013; Zhang L. Y. et al., 2015), peach (Guo et al., 2013), pear (Xiao et al., 2012), and litchi (Feng et al., 2015). Importantly, it was also reported that ALA significantly increased fruit interior quality (Gao et al., 2013; Zhang L. Y. et al., 2015). Furthermore, ALA is readily biodegradable and has no adverse effects on animals and humans (Perez et al., 2013). Therefore, ALA can simultaneously improve fruit coloration and fruit interior quality without any detrimental effects, suggesting great application prospect in fruit production.

Red coloration in various plant tissues is predominantly caused by anthocyanin, which accumulates as granules in the vacuole (Bae et al., 2006). This pigment belongs to the diverse group of ubiquitous secondary metabolites collectively known as flavonoids. In plants, two categories of genetic control relate to anthocyanin accumulation. One category is the biosynthetic genes that encode enzymes required for anthocyanin biosynthesis, including chalcone synthase (CHS), chalcone isomerase (CHI), flavanone-3-hydroxylase (F3H), dihydroflavonol-4-reductase (DFR), leucoanthocyanidin dioxygenase (LDOX), and UDP-glycose: flavonoid 3-O-glucosyl- transferase (UFGT) (An et al., 2015). All of these six genes have been isolated in various plants and their transcription levels are positively correlated with anthocyanin concentration (Han et al., 2010; Feng et al., 2013). Another category is regulatory genes that influence the intensity and pattern of anthocyanin biosynthetic genes. In this category, most studies on the regulation of anthocyanins have focused on transcription factors of R2R3-MYB, basic helix-loop-helix (bHLH), and WD40 classes. These three regulators can form a MYB-bHLH-WD40 protein complex that binds to promoters and then induces transcription of anthocyanin biosynthetic pathway genes. In recent two decades, additional potential regulators have also been reported in model plant *Arabidopsis thaliana* to affect anthocyanin synthesis, including PHYTOCHROME-INTERACTING FACTOR 3 (PIF3), LONG HYPOCOTYL 5 (HY5), CONSTITUTIVELY PHOTOMORPHOGENIC 1 (COP1), WRKY, WIP domain, MADS-box domain, NAC (NAM, ATAF, CUC), Jasmonate ZIM-domain (JAZ), and the SQUAMOSA promoter-binding protein-like (SPL) (Zhou et al., 2015).

Several studies have been conducted to investigate the regulatory mechanisms behind anthocyanin accumulation in apple. Conserved *MYB, bHLH*, and *WD40* genes in the apple that are homologs of *Arabidopsis* MYB-bHLH-WD40 protein complex have been demonstrated to be responsible for the accumulation of anthocyanins (Takos et al., 2006; An et al., 2012; Xie et al., 2012). Likewise, new regulators involved in anthocyanin biosynthesis were identified in apple fruits. For example, MdCOP1 has been demonstrated to be involved in the ubiquitination and degradation of the MdMYB1 protein under dark conditions (Li et al., 2012) and MdJAZ2 has been proposed to be involved in the regulation of anthocyanin accumulation during the response of apple fruits to jasmonate (An et al., 2015). Since the regulatory mechanism modulates anthocyanin biosynthesis is highly conserved in higher plants, more research is necessary to develop the anthocyanin regulation network in apple. Research on ALA promoting anthocyanin accumulation in apple fruits has been linked to up-regulating anthocyanin biosynthetic genes and regulatory genes *MYB, bHLH*, and *WD40* (Xie et al., 2013). However, little information is available regarding special regulative effects of ALA on fruit skin and its regulatory mechanisms remain unknown. Current knowledge about the function of ALA on fruit is derived from research on some physiological aspects of fruit growth and ripening. Therefore, an overall molecular framework is needed for better understanding the ALA-associated fruit coloration.

Proteomic and transcriptomic techniques are often used to investigate the molecular mechanisms involved in complex traits. To make a comprehensive understanding of ALA-stimulated fruit coloration, integrated proteomic, and transcriptomic techniques were employed in this study. We identified and analyzed ALA-induced various changes at protein and mRNA levels using gel-free and two-dimensional gel electrophoresis (2-DE) gel-based proteomic techniques and suppression subtractive hybridization (SSH). Based on the results of proteomics and SSH, a candidate biomarker *MdMADS1* was selected to explore the molecular mechanism underlying ALA-induced anthocyanin accumulation. Our data offers new molecular evidence elucidating the regulatory mechanism of fruit coloration by ALA, and provides a valuable reference for further research on anthocyanin accumulation in apple fruits.

## Materials and methods

### Fruit source and apple flesh calli induction

Fruits were collected from apple (*Malus* × *domestica* Borkh. cv. Fuji) trees at commercial apple orchards of eastern China, Fengxian County in Jiangsu Province. All fruits were bagged with paper-bags in late May, debagged in early October, and the fruits were commercially harvested in late October. In this study, the debagged fruits which were collected from eight trees were harvested in early October (at onset of fruit coloration) and immediately transported to the laboratory for two different treatments. Solutions containing 0.01% Tween-20 alone (control), or with 200 mg/L ALA (treatment) were sprayed to the surface of debagged fruits. The fruits were then transferred into growth chamber with 150 μmol m^−2^ s^−1^ photon flux density (PFD) at 22°C and sampled at 0, 6, 12, 24, 36, 48, and 72 h of light exposure. At each of the sampling points, skins from 15 different fruits were collected and divided randomly into two groups. One group of skin samples was used for measurements of anthocyanin content, and the other was stored at −80°C for RNA and protein isolation after being frozen in liquid nitrogen. Since the time course of anthocyanin accumulation in apple skin showed that the promotion of ALA on anthocyanin accumulation initiated after 24 h light irradiation (See the “Results”), to identify the early upstream regulators of anthocyanin biosynthesis induced by ALA, we chose skins of apples that exposed to light for 24 h for the proteomics and SSH analysis.

Calli from “Fuji” fruit flesh were induced on Murashige and Skoog medium containing 1 mg/L 6-benzylaminopurine (BAP) and 1 mg/L 2,4-dichloropheno-xyacetic acid (2,4-D) at 25°C in the dark, and subcultured at 14-day intervals. For ALA treatment, the calli were transferred to medium containing 100 mg/L ALA. To induce anthocyanin accumulation, transgenic calli were cultured in a culture room under 100 μmol m^−2^ s^−1^ PFD at 25°C for 4 days, and then collected for determination of anthocyanin content and RNA isolation.

### Measurement of anthocyanin content

Anthocyanin content was extracted using 1% (v/v) HCl-methanol for 24 h at room temperature in the dark. The extracts were centrifuged at 15,000 g for 15 min, and the absorbance at 530 nm was then measured with a spectrophotometer. The amount of anthocyanin was expressed as nmol of cyanidin-3-galactoside per g of the sample using a molar extinction coefficient of 3.43 × 10^4^ (Ubi et al., 2006). Mean values were obtained from five independent replicates.

### Proteomic analysis by 2-DE

Protein extraction of apple skin was performed using phenol-based method (Deytieux et al., 2007). The final pellet was dissolved in a solution containing 7 M urea, 2 M thiourea, 4% (w/v) 3-[(3-cholamidopropyl) dimethylammonio]-1- propanesulfonate (CHAPS), 1% (w/v) dithiothreitol (DTT), and 0.5% (v/v) immobilized pH gradient (IPG) buffer (pH 4–7; GE Healthcare, USA). The protein concentration was quantified according to the method suggested by Bradford (1976), using bovine serum albumin as standard.

For 2-DE, protein samples (1 mg) were brought to 450 μL of isoelectric focusing (IEF) rehydration solution (7 M urea, 2 M thiourea, 4% (w/v) CHAPS, 1% (w/v) DTT, 0.5% (v/v) IPG buffer, and 0.01% bromphenol blue). The whole volume was transferred into a well of the Immobiline DryStrip Reswelling Tray and IPG strips (24 cm, pH 4–7; GE Healthcare) were rehydrated overnight at 20°C. The strips were then loaded onto an Ettan IPGphor 3 instrument (GE Healthcare), and IEF was performed according to the following steps: 100 V for 1 h, 500 V for 1 h, followed by 8 h gradient from 1000 to 10000 V, and finally focused for 65,000 Vh at 10,000 V. The maximum current per strip was set at 50 μA. After two-step equilibration, the IPG strips were loaded on a 12% w/v sodium dodecyl sulfate-polyacrylamide gel electrophoresis (SDS-PAGE) gels using the Ettan Daltsix system (GE Healthcare). The gels were run at 150 V until the dye front reached the bottom of the gel. The gels were visualized using the Coomassie Brilliant Blue G-250 stain and scanned using Image Scanner software (GE Healthcare). Afterward, gel images were processed using the PDQuest 2-DE analysis software (Version 8.0.1, Bio-Rad, USA) in a three-step manner: spot detection, volumetric quantification, and matching. Differences in protein content were analyzed using a *t*-test and calculated as the fold ratio in three biological replicates with two technical replicates. A threshold of *P* < 0.05 and fold change of >1.5 or <0.67 was used to identify significant differentially expressed protein spots.

Spots from 2-DE were excised from the gel and digested with trypsin. Then, samples were re-suspended with 5 μL 0.1% TFA followed by mixing in 1:1 ratio with a matrix consisting of a saturated solution of α-cyano-4-hydroxy-trans-cinnamic acid in 50% ACN containing 0.1% TFA. One microliter mixture was spotted on a stainless steel sample target plate. Peptide mass spectrometry (MS) and MS/MS were performed on an ABI 5800 MALDI-TOF/TOF Plus mass spectrometer (Applied Biosystems, USA). Data were acquired in the positive MS reflector using a CalMix5 standard to calibrate the instrument (ABI5800 Calibration Mixture). Both the MS and MS/MS data were integrated and processed using GPS Explorer V3.6 software (Applied Biosystems, USA) with default parameters. Based on the combined MS and MS/MS spectra, proteins were successfully identified based on 95% or higher confidence interval of their scores in MASCOT V2.3 search engine (Matrix Science Ltd, London, U.K.), using the following search parameters: the apple expressed sequence tag (EST) database (32,768 entries, Jan. 14th 2014) downloaded from the Genome Database for Rosaceae (GDR) (https://www.rosaceae.org/species/malus/malus_x_domestica/genome_v1.0); trypsin as the digestion enzyme; one missed cleavage site; fixed modifications of Carbamidomethyl (C); partial modifications of Acetyl (Protein N-term), Deamidated (NQ), Dioxidation (W), Oxidation (M); 100 ppm for precursor ion tolerance, and 0.5 Da for fragment ion tolerance.

The functional annotation of the identified proteins was based on UniProt, GDR, NCBInr protein database, and the literature.

### Proteomic analysis by label-free

For shotgun analysis, apple skin proteins (100 μg) dissolved in 6 M urea and 50 mM Tris-HCl (pH 8.0) were reduced by added 1 M DTT until at final concentration of 4 mM for 1 h at 60°C, and then added with 1 M iodoacetamide until at a final concentration of 25 mM for 45 min at 25°C in the dark. The 6 M urea was removed by ultrafiltration in case it influenced digestion. Proteins were dissolved in 50 mM NH_4_HCO_3_ (pH 7.8) and then treated with trypsin (2 μg, Promega, USA) at 37°C for 12 h. Finally, protein desalted using a C18 column (Empore) and freeze-dried before sample injection.

A liquid chromatography-MS (LC-MS) system consisting of a Dionex Ultimate 3000 nano-LC system (nano UHPLC, Sunnyvale, CA, USA), connected to a linear quadrupole ion trap Orbitrap (LTQ Orbitrap XL) mass spectrometer (Thermo Electron, Bremen, Germany), and equipped with a nanoelectrospray ion source. For LC separation, an Acclaim PepMap 100 column (C18, 3 μm, 100 Å) (Dionex, Sunnyvale, CA, USA) capillary with a 15 cm bed length was used with a flow rate of 300 nL/min. Two solvents, A (0.1% formic acid) and B (aqueous 80% acetonitrile in 0.08% formic acid), were used to elute the peptides from the nanocolumn. The gradient went from 5 to 40% B in 32 min and from 40 to 95% B in 1 min, with a total run time of 60 min. The mass spectrometer was operated in the data-dependent mode so as to automatically switch between Orbitrap-MS and LTQ-MS/MS acquisition. Electrospray voltage and the temperature of the ion transfer capillary were 2.2 kV and 200°C, respectively. Survey full scan MS spectra (from m/z 350 to 1800) were acquired in the Orbitrap with a resolution *r* = 60,000 at m/z 400, allowing the sequential isolation of the top ten signal intensity ions for collision-induced dissociation at a collision energy of 35 V. A dynamic exclusion mode was enabled to exclude the previously selected ions during the repeated cycle of 60 s. The external mass calibration of the Orbitrap was performed once every 3 days to ensure a working mass accuracy <5 ppm. For each run, 1.5 μg of the digest was injected on a reverse-phase C18 column.

The obtained MS/MS spectra were searched against the apple EST database using SEQUEST algorithm in Proteome Discoverer 1.3 software (Thermo Scientific, San Jose, CA, USA). Search results were filtered for a False Discovery rate of 1% employing a decoy search strategy utilizing a reverse database.

For quantitative proteome analysis, four MS raw files from each group were analyzed using SIEVE software (Version 2.0, Thermo Scientific, USA). Eight MS raw files were performed the SIEVE experimental workflow of “two sample differential analysis,” where ALA–treated samples were compared to control samples. For the alignment step, the chromatographic peaks detected by Orbitrap were aligned by their retention time (± 2.5 min) and mass (± 0.02 unit) among all sample runs. After alignment, the feature detection and integration (or framing) process were performed using the MS level data with a feature called “Frames from MS2 Scans” only. The parameters consisted of a frame m/z width of 10 ppm and a retention time width of 2.5 min. Then, the peak integration was performed for each frame and these values were used for statistical analysis. Next, peptide sequences obtained from the database search were imported into SIEVE. A frame filter rule which was defined as “PRRoot > 0 and GoodID > 0 and CV_ALA treatment <25 and CV_control <25” was applied to obtain confident overall protein ratio. Peptides were grouped into proteins and a protein ratio and *P*-value were calculated. Only proteins observed in all two groups were quantified. The quantified proteins were considered as significantly different expressed proteins if they matched at least two peptides, and changed over 1.5-fold cutoff (ratio above 1.5 or below 1/1.5) with *P*-value < 0.05.

### Suppression subtractive hybridization (SSH)

Total RNA was isolated from apple skins by CTAB-LiCl methods (Jaakola et al., 2001). Isolation of poly A^+^ RNA from total RNA was performed by PolyATtract mRNA Isolation Systems Kit (Promega, USA) according to the manufacturer's instructions. The integrity of RNA was ascertained by electrophoresis on 1% agarose gel. The concentration of total RNA and poly A^+^ RNA was measured by Nanodrop 2000 spectrophotometer (Thermo Scientific, USA).

The SSH library was carried out using the PCR-Select™ cDNA Subtraction Kit (Clontech, USA) according to the manufacturer's instructions, starting with 2 μg poly A^+^ RNA from the tester (ALA) and driver (control) samples. After checking subtraction efficiency, subtracted second PCR products were cloned into the pMD-19T vector (Takara, Dalian, China) and then transformed into Trans1-T1 phage resistant chemically competent cells (TransGen Biotech, China). Subsequently, white colonies from Luria-Bertani (LB) solid medium plates containing ampicillin, 5-bromo-4-chloro-3-indolyl-β-D-galacto-pyranoside (X-Gal), and isopropyl-β-D-1- thiogalactopyranoside (IPTG) were selected for colony polymerase chain reaction (PCR). The PCR amplification used the primer set of Nested PCR primer 1 and Nested PCR primer 2R provided along the SSH kit. Then, clones which PCR products were single inserts and longer than 100 bp insertion fragment size were sequenced with the universal M13 sequencing primer. After removing vector and adaptor sequences from the raw EST sequences, the resulting ESTs were assembled into unigenes using iAssembler (Zheng et al., 2011). Annotation and function analysis of the resulting unigenes were compared against the apple EST database.

### Transfection of apple fruit calli by agroinfiltration

In order to overexpress *MdMADS*1 (Accession No. U78947), the open reading frame was amplified by PCR from cDNA of Fuji apple fruits skin with primers MdMADS1(OE)-F and MdMADS1(OE)-R (Supplementary Table S1), followed by ligation to the linearized plant transformation vector pBI121 based on homologous recombination method using the NovoRec® PCR Seamless Cloning Kit (Novoprotein Scientific Inc, China).

To silence *MdMADS1*, a 259 base pair (bp) fragment including 58 bp sequence homologous to the RNA-interference (RNAi) vector pHELLSGATE2 and 201 bp of *MdMADS1* coding sequence was amplified by RT-PCR using primers MdMADS1(i)-F and MdMADS1(i)-R (Supplementary Table S1). Amplified fragments were transferred to the RNAi vector through Gateway BP reaction generating RNAi construct in which the sense and antisense *MdMADS1* RNA sequences would be linked in tandem separated by the PDK intron.

The resulting recombinant plasmids and empty plasmids were transformed into *Agrobacterium* strain EHA105. One single separated colony containing the target gene was grown at 28°C in LB broth supplemented with appropriate antibiotics. When the optical density at 600 nm (OD600) of the culture liquid reached approximately 0.5, *Agrobacterium* cells were harvested and resuspended in a modified MacConkey agar (MMA) medium (Murashige and Shoog salts, 10 mM morpholine ethane sulphonic acid pH 5.6, 20 g/L sucrose). The fresh calli were soaked into the *Agrobacterium* solution for 15 min. The calli were co-cultured on Murashige and Skoog medium containing 1 mg/L 2, 4-D and 1 mg/L BAP for 2 d at 25°C in the dark. Subsequently, the calli were washed 5 times with sterile water and transferred to MS medium supplemented with 250 mg/L of carbenicillin and 30 mg/L kanamycin for transgenic selection.

### Quantitative real-time reverse transcription-PCR

The cDNA synthesis was performed using the PrimeScript First-Stand cDNA Synthesis Kit (TaKaRa Bio, China). Quantitative real-time reverse transcription-PCR (qRT-PCR) was performed with SYBR® Premix Ex Taq™ (TaKaRa Bio, China) as described by the manufacturer's instructions. Using specific primers (Supplementary Table S1), the transcript levels were calculated using the 2^−ΔΔCT^ method (Livak and Schmittgen, 2001), where *MdACTIN* gene was used as an internal reference. In this study, the relative expression levels of corresponding genes were presented as values relative to corresponding indicated samples. Data were derived from 3 independent replicates.

### Statistical analysis

All data were obtained from at least three independent experiments. Statistical and correlation analysis was performed using SPSS statistical computer package (version 19.0 SPSS Inc. Chicago. IL). Data was compared with the control or among treatments by analysis of variance (ANOVA) to discriminate significant differences at *P* < 0.05 followed by least significant difference tests (LSD).

## Results

### ALA promoted anthocyanin accumulation in the skin of debagged apple fruits

**Figure 1:**
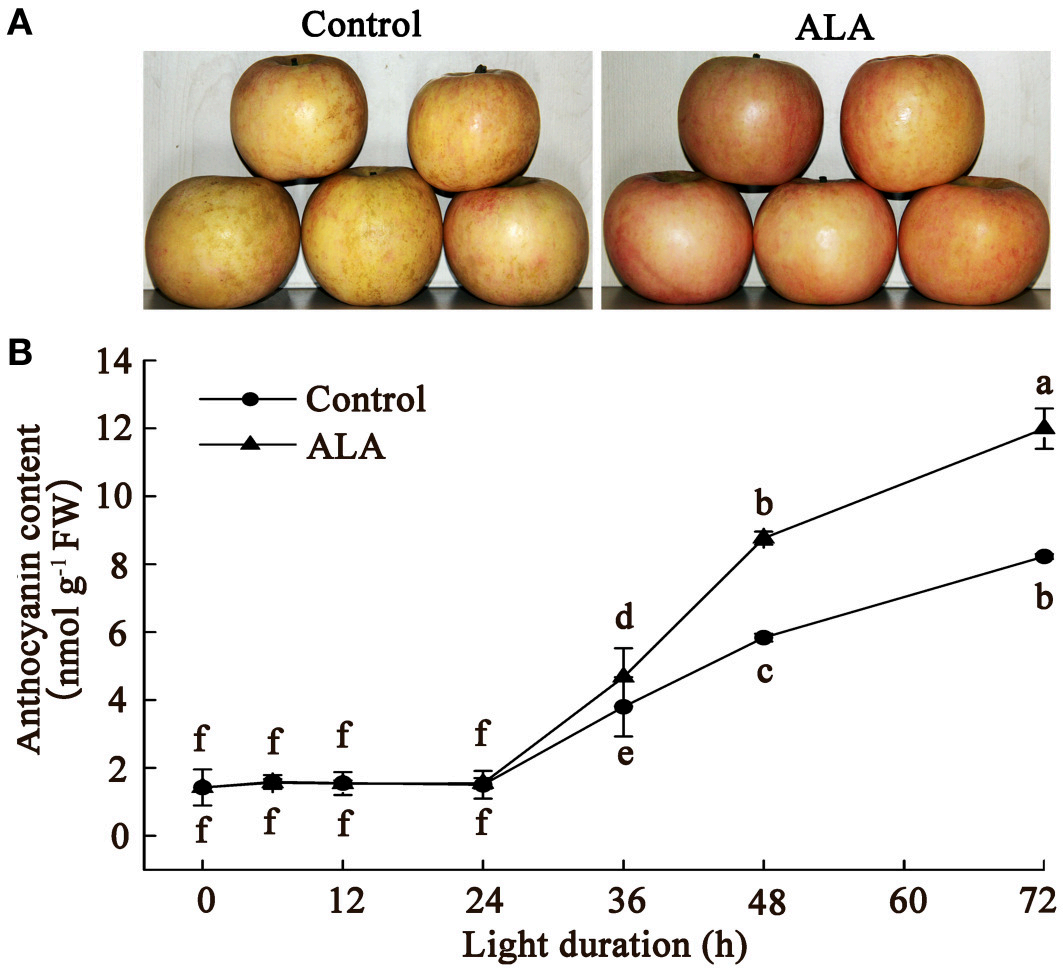
**ALA induces anthocyanin accumulation in apple skin**. The bagged fruits from Fuji apple trees were collected in early October and immediately transported to the laboratory for 200 mg/L ALA treatment and water treatment (control). Then, fruits coloration was induced in a growth chamber with 150 μmol m^−2^ s^−1^ photon flux density at 22°C. **(A)** A photo displays the color difference between ALA treatment and the control in apple skin at 72 h light duration. **(B)** Time courses of anthocyanin accumulation after ALA application. The different small letters represent significant differences at *P* = 0.05 level.

To confirm the effect of ALA on apple fruit coloration and provide more detailed information on this process, we investigated the time course of anthocyanin accumulation in apple skin after ALA treatment. Results showed that ALA treatment notably promoted fruit coloration within 72 h of light irradiation (Figure 1A). Anthocyanin determination showed that ALA did not stimulate anthocyanin accumulation within 24 h, but significantly increased anthocyanin content after 24 h, compared with the control (Figure 1B). Anthocyanin content in ALA-treated apple skin was about 23, 50, and 45%, respectively, higher than that in control at 36, 48, and 72 h. These results indicated that ALA-induced upstream regulatory factors of anthocyanin accumulation probably function before 36 h. Therefore, to identify the early upstream regulators of anthocyanin biosynthesis induced by ALA, skins of apples that exposed to light for 24 h were used for the following proteomics and SSH analysis.

### Protein identification using gel-based and gel-free proteomics

**Figure 2:**
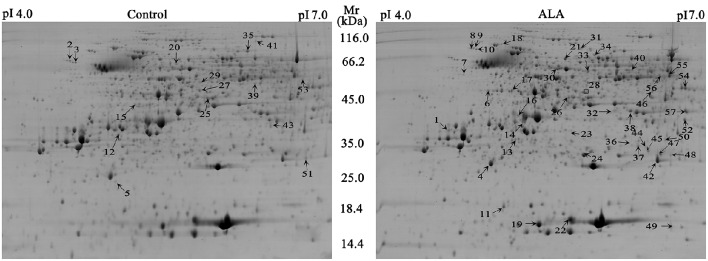
**Representative 2-DE gel images of ALA treatment and the control in apple skin**. Nearly 500 resolved spots were detected with Mr ranging from 116.0 to 14.4 kDa and pI ranging from 4.0 to 7.0. A total of 57 spots (marked with arrow and number) were identified as differentially expressed and of these, 15 proteins were down-regulated (left), whereas 42 proteins were up-regulated in ALA treatment (right). One spot was unsuccessfully identified (marked with square).

To explore the mechanism how ALA regulated anthocyanin accumulation, we measured changes in the abundance of proteins and compared them between control and ALA-treated fruit skin using 2-DE gel-based proteomics. Analysis of the 2-DE pattern revealed that nearly 500 resolved spots were detected after ignoring very faint spots and spots with undefined shapes and areas (Figure 2). To analyze ALA-responsive proteins, the changes in spot intensity between ALA-treated and control were quantified by PDQuet software. Quantitative analysis of spot intensity by integration of the staining signal for each gel revealed that levels of 57 proteins changed in an ALA-dependent manner (ratio > 1.5 or ratio <0.67) in three independent replications. Using MALDI-TOF/TOF MS/MS, 56 protein spots representing 50 differently expressed proteins were then successfully identified according to the GDR database (Supplementary Table S2).

To obtain a more comprehensive understanding of proteins affected by ALA, we further performed label-free shotgun proteomics of apple skin. Data acquired by nano-ESI-MS/MS on a Q-Exactive spectrometer were processed with SIEVE software to reveal up- or down-regulated proteins by ALA. A total of four runs per group were analyzed and the quality of the alignment of the chromatographic peaks between ALA treatment group and control group was high (Supplementary Figure S1). Significant differences in protein abundance were considered with a ratio of ALA-treated/control higher than 1.5 or lower than 0.67 (*P* < 0.05). As a result, a total of 47 proteins were identified (Supplementary Table S3).

**Figure 3:**
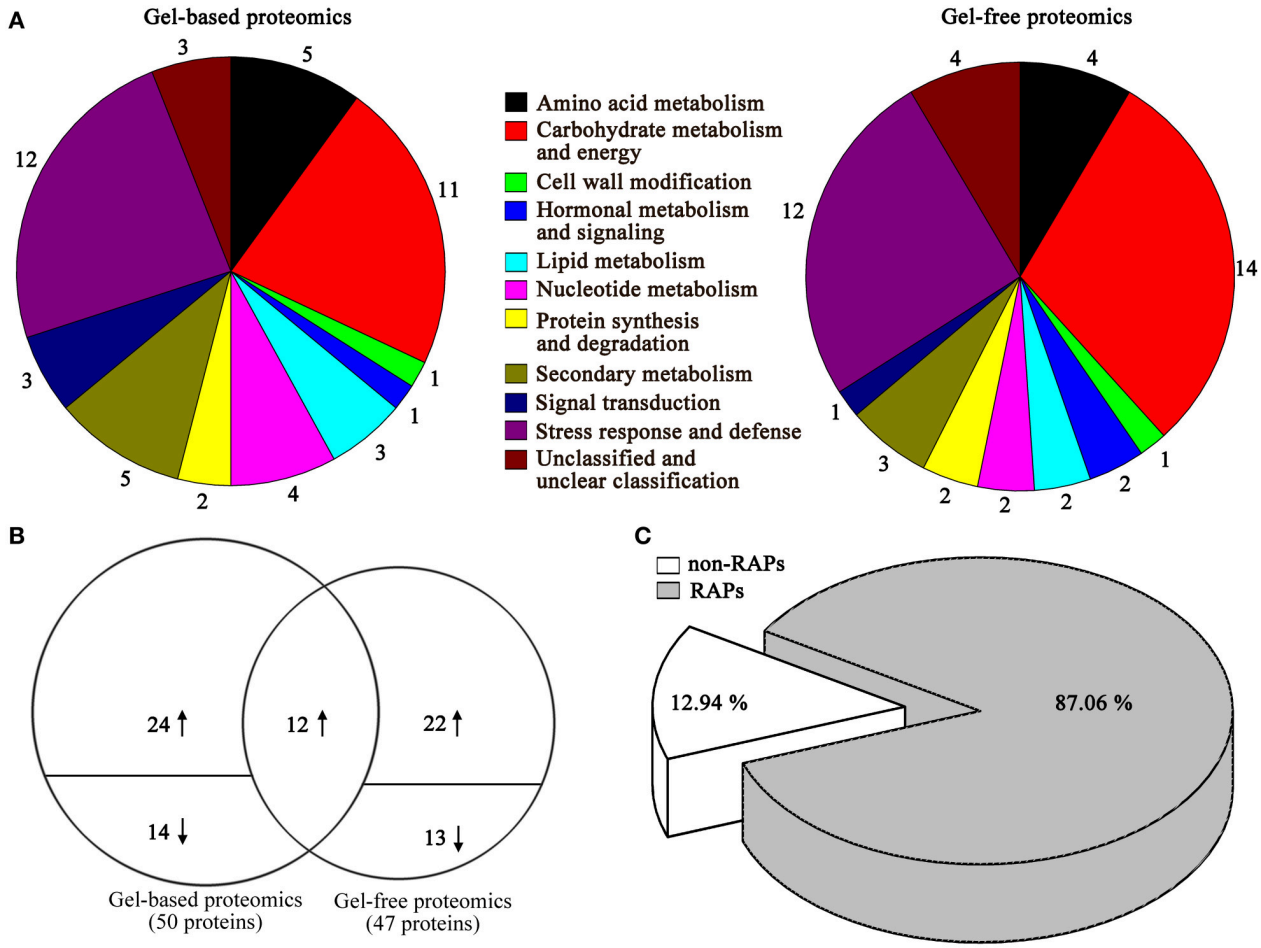
**Proteomic analyses of differentially expressed proteins between ALA treatment and the control in apple skin using gel-free and gel-based techniques**. **(A)** Primary functional distribution of 50 and 47 regulated gene products as identified by gel-free and gel-based techniques. **(B)** Venn diagram of the distribution of differentially expressed proteins identified by gel-free and gel-based techniques. The number above or below the horizontal line in each portion indicated the number of up-regulated or down-regulated proteins. The overlapping regions indicated the number of proteins commonly identified by the two proteomics techniques. **(C)** Further classification was carried out to identify proteins related to previous studies orthologous ripening associated proteins (RAPs).

The identified proteins by gel-based and gel-free proteomics were further categorized into different classes (Figure 3A), where the 50 proteins identified using gel-based proteomics were mainly involved in stress response and defense, carbohydrate metabolism and energy, amino acid metabolism, nucleotide metabolism, lipid metabolism, nucleotide metabolism, and secondary metabolism. Similarly, the 47 proteins identified using gel-free proteomics were predominantly related to stress response and defense, carbohydrate metabolism and energy, amino acid metabolism and secondary metabolism.

Combined the results of gel-based and gel-free proteomics, a total of 85 differently expressed proteins were identified (Table 1, Figure 3B). Among these proteins, 72.82% of changed proteins were up-regulated by ALA, and 12 proteins were commonly identified by the two proteomics techniques (Supplementary Table S4). Moreover, among the total ALA-responsive proteins, about 87.06% of changed proteins are related to orthologous fruit ripening associated genes (Figure 3C), indicating that ALA might increase anthocyanin accumulation by regulating fruit ripening process.

**Table 1:** **Differently expressed proteins obtained by 2-DE and label-free analysis in ALA-treated apple skin**.

**No**.	**Regulated^a^**	**Accession No**.	**Annotation**	**Method**	**Ripening associated references**
**AMINO ACID METABOLISM**					
1	−	MDP0000663130	Acireductone dioxygenase	2-DE	n/a
2	−	MDP0000806502	4-hydroxyphenylpyruvate dioxygenase	2-DE	Apple (Zhang Z. et al., 2015)
3	−	MDP0000188304	Aminoacylase-1	2-DE	Grape (Fraige et al., 2014)
4	+	MDP0000301987	Ketol-acid reductoisomerase	2-DE	Peach (Prinsi et al., 2011)
5	+	MDP0000148984	Methylthioribose kinase	2-DE	Tomato (Kushad et al., 1985)
6	+	MDP0000668552	Glutamine amidotransferase-like Class I superfamily protein	label-free	n/a
7	−	MDP0000284588	Glutamate decarboxylase	label-free	Tomato (Gallego et al., 1995) Citrus (Liu et al., 2014)
8	−	MDP0000239328	Methylmalonate-semialdehyde dehydrogenase	label-free	Apple (Zhang Z. et al., 2015)
9	−	MDP0000147916	Serine acetyltransferase	label-free	n/a
**CARBOHYDRATE METABOLISM AND ENERGY**					
10	+	MDP0000234480	Transaldolase	2-DE	Kiwifruit (Minas et al., 2012)
11	+	MDP0000147610	F-type ATPases	2-DE label-free	Apricot (D'Ambrosio et al., 2013) Grape (Giribaldi et al., 2007; Martínez-Esteso et al., 2013)
12	+	MDP0000769597	Pyruvate dehydrogenase E1 subunit-β	2-DE	Date palm (Marondedze et al., 2014)
13	+	MDP0000316685	Transaldolase	2-DE	Kiwifruit (Minas et al., 2012)
14	+	MDP0000248012	Vacuolar proton pump subunit A	2-DE	Papaya (Huerta-Ocampo et al., 2012) Grape (Giribaldi et al., 2007; Martínez-Esteso et al., 2013)
15	+	MDP0000300513	Vacuolar proton ATPase subunit C	2-DE	Grape (Giribaldi et al., 2007; Martínez-Esteso et al., 2013; Fraige et al., 2014)
16	+	MDP0000198482	Glyceraldehyde-3-phosphate dehydrogenase	2-DE	Apple (Zheng et al., 2013)
17	+	MDP0000249227	Soluble inorganic pyrophosphatase	2-DE label-free	Apricot (D'Ambrosio et al., 2013) Grape (Martínez-Esteso et al., 2013)
18	+	MDP0000755275	Aldose 1-epimerase	2-DE	Grape (Martínez-Esteso et al., 2013)
19	+	MDP0000273688	Fructose-bisphosphate aldolase	2-DE label-free	Kiwifruit(Minas et al., 2012) Grape (Martínez-Esteso et al., 2013; Fraige et al., 2014)
20	+	MDP0000179036	Enolase	2-DE	Kiwifruit (Minas et al., 2012) Grape (Negri et al., 2008; Martínez-Esteso et al., 2013)
21	+	MDP0000297664	Putative mitochondrial 2-oxoglutarate/malate carrier protein	label-free	n/a
22	+	MDP0000298613	Ubiquinol-cytochrome c reductase complex 14 kDa protein	label-free	n/a
23	+	MDP0000442105	LYR family of Fe/S cluster biogenesis protein	label-free	n/a
24	+	MDP0000321341	Pyrophosphate: fructose 6-phosphate 1-phosphotransferase	label-free	Tomato (Wong et al., 1990)
25	+	MDP0000796883	Adenine nucleotide translocator	label-free	Tomato (Kumar et al., 2015)
26	+	MDP0000217005	Transketolase	label-free	Kiwifruit (Minas et al., 2012)
27	+	MDP0000527995	Glyceraldehyde-3-phosphate dehydrogenase	label-free	Apple (Zheng et al., 2013)
28	−	MDP0000153379	L-arabinokinase	label-free	n/a
29	−	MDP0000183725	Pyruvate kinase	label-free	Apple and tomato (Janssen et al., 2008)
30	−	MDP0000597996	Ribulose-1-5-bisphosphate carboxylase	label-free	Apple (Zheng et al., 2013)
31	−	MDP0000268037	NADP-dependent malic enzyme	label-free	Apple (Shi et al., 2014)
**CELL WALL MODIFICATION**					
32	+	MDP0000416548	β-Galactosidase	2-DE	Apple (Shi et al., 2014)
33	+	MDP0000269483	Xyloglucan endotransglucosylase/hydrolase protein 6	label-free	Grape (Negri et al., 2008)
**HORMONAL METABOLISM AND SIGNALING**					
34	+	MDP0000195885	1-aminocyclopropane-1-carboxylate oxidase 1	2-DE label-free	Apple (Shi et al., 2014)
35	+	MDP0000219737	Ethylene receptor 2	label-free	Apple (Li and Yuan, 2008)
**LIPID METABOLISM**					
36	+	MDP0000333058	Acetyl-CoA carboxyltransferase β-subunit	2-DE	n/a
37	−	MDP0000764262	3,4-dihydroxy-2-butanone kinase	2-DE	n/a
38	−	MDP0000300217	Phospholipase D alpha	2-DE	Strawberry (Yuan et al., 2006)
39	−	MDP0000450991	Lipoxygenase	label-free	Tomato (Qin et al., 2012)
40	+	MDP0000940078	Plant lipid transfer protein	label-free	Apple and tomato (Janssen et al., 2008)
**NUCLEOTIDE METABOLISM**					
41	+	MDP0000121897	Adenine phosphoribosyltransferase	2-DE label-free	Apple (Shi et al., 2014)
42	−	MDP0000225318	Adenosine kinase	2-DE	Grape (Negri et al., 2008)
43	+	MDP0000322880	Nucleoside diphosphate kinase	2-DE label-free	Kiwifruit (Minas et al., 2012) Papaya (Huerta-Ocampo et al., 2012)
44	−	MDP0000165865	Uridine 5'-monophosphate synthase	2-DE	n/a
**PROTEIN SYNTHESIS AND DEGRADATION**					
45	−	MDP0000146975	Glycyl-tRNA synthetase 1	2-DE	n/a
46	−	MDP0000014145	Proteasome subunit β type-7	2-DE	Apple (Shi et al., 2014)
47	−	MDP0000256937	40S ribosomal protein S3-3-like	label-free	Apple (Zhang Z. et al., 2015)
48	−	MDP0000616695	60S ribosomal protein L11 isoform X1	label-free	Tomato (Kumar et al., 2015)
**SECONDARY METABOLISM**					
49	+	MDP0000609966	Polyphenol oxidase	2-DE label-free	Apricot (D'Ambrosio et al., 2013) Grape (Negri et al., 2008; Fraige et al., 2014)
50	+	MDP0000052862	UDP-glucose: anthocyanidin 3-O-glucosyltransferase	2-DE label-free	Apricot (D'Ambrosio et al., 2013) Tomato (Kumar et al., 2015)
51	+	MDP0000221498	Polyphenol oxidase	2-DE	Apricot (D'Ambrosio et al., 2013) Grape (Negri et al., 2008; Fraige et al., 2014)
52	−	MDP0000269612	Cinnamoyl-CoA reductase	2-DE	n/a
53	+	MDP0000699845	Polyphenol oxidase	2-DE	Apricot (D'Ambrosio et al., 2013) Grape (Negri et al., 2008; Fraige et al., 2014)
54	+	MDP0000615956	4-coumarate-CoA ligase	label-free	Apple (Zhang Z. et al., 2015)
**SIGNAL TRANSDUCTION**					
55	+	MDP0000270640	14-3-3 protein 7-like	2-DE	Apple (Shi et al., 2014)
56	+	MDP0000376563	Protein phosphatase 2c-like protein	2-DE	Citrus (Wu et al., 2014)
57	+	MDP0000166687	14-3-3 protein	2-DE	Apple (Shi et al., 2014)
58	+	MDP0000325949	14-3-3 protein family	label-free	Apple (Shi et al., 2014)
**STRESS RESPONSE AND DEFENSE**					
59	+	MDP0000298502	Heat shock protein 70	2-DE	Kiwifruit (Minas et al., 2012) Papay (Huerta-Ocampo et al., 2012; Nogueira et al., 2012)
60	+	MDP0000416706	Heat shock protein 70	2-DE	Kiwifruit (Minas et al., 2012) Papay (Huerta-Ocampo et al., 2012; Nogueira et al., 2012)
61	+	MDP0000303430	Heat shock protein 90	2-DE	Apricot (D'Ambrosio et al., 2013) Grape (Negri et al., 2008)
62	+	MDP0000246775	Thaumatin-like protein 1a	2-DE label-free	Apple (Shi et al., 2014)
63	+	MDP0000277802	MLP-like protein 329	2-DE label-free	Apple (Shi et al., 2014)
64	+	MDP0000103621	Major allergen mal d 1	2-DE	Apple (Shi et al., 2014)
65	+	MDP0000199034	L-ascorbate peroxidase	2-DE label-free	Apple (Shi et al., 2014)
66	+	MDP0000248823	L-aascorbate peroxidase 6	2-DE	Apple (Shi et al., 2014)
67	−	MDP0000287459	Aldo/keto reductase	2-DE	Papaya (Huerta-Ocampo et al., 2012) Grape (Martínez-Esteso et al., 2013)
68	+	MDP0000096349	Glutathione S-transferase	2-DE label-free	Apple (Shi et al., 2014)
69	+	MDP0000868045	Abscisic acid response protein	2-DE	Apple (Shi et al., 2014)
70	+	MDP0000261821	Monodehydroascorbate reductase	2-DE	Tomato (Kumar et al., 2015)
71	+	MDP0000519575	Peroxiredoxin	label-free	Apple (Zhang Z. et al., 2015)
72	+	MDP0000913598	Glutathione peroxidase	label-free	Apricot (D'Ambrosio et al., 2013) Tomato (Qin et al., 2012)
73	+	MDP0000320612	Peroxiredoxin	label-free	Apple (Shi et al., 2014)
74	+	MDP0000253074	Abscisic acid stress ripening protein homolog	label-free	Apple (Shi et al., 2014)
75	+	MDP0000280265	Acidic endochitinase	label-free	Apricot (D'Ambrosio et al., 2013)
76	+	MDP0000452572	Universal stress protein	label-free	Apple (Shi et al., 2014)
77	+	MDP0000288293	Major allergen Pru ar 1	label-free	Apple (Shi et al., 2014)
78	−	MDP0000770493	Dehydrin-like protein	label-free	Peach (Prinsi et al., 2011)
**UNCLASSIFIED AND UNCLEAR CLASSIFICATION**					
79	−	MDP0000201559	Plasma membrane-associated cation-binding protein 1-like isoform 2	2-DE	n/a
80	−	MDP0000296243	NAD(P)-binding Rossmann-fold superfamily protein	2-DE	n/a
81	−	MDP0000170439	Uncharacterized protein	2-DE	n/a
82	−	MDP0000543784	Uncharacterized protein	label-free	n/a
83	+	MDP0000279955	Uncharacterized protein	label-free	n/a
84	+	MDP0000251031	Uncharacterized protein	label-free	n/a
85	−	MDP0000250284	Uncharacterized protein	label-free	n/a

a Preoteins were up-regulated (+) or down- regulated (−) in ALA-treated apple skin compred to control.

The detailed information on the fold ratio (ALA/control) and P-value were exhibited in Supplementary Tables S2 and S3.

### Screening of ALA-induced genes from apple skins by suppression subtractive hybridization

**Figure 4:**
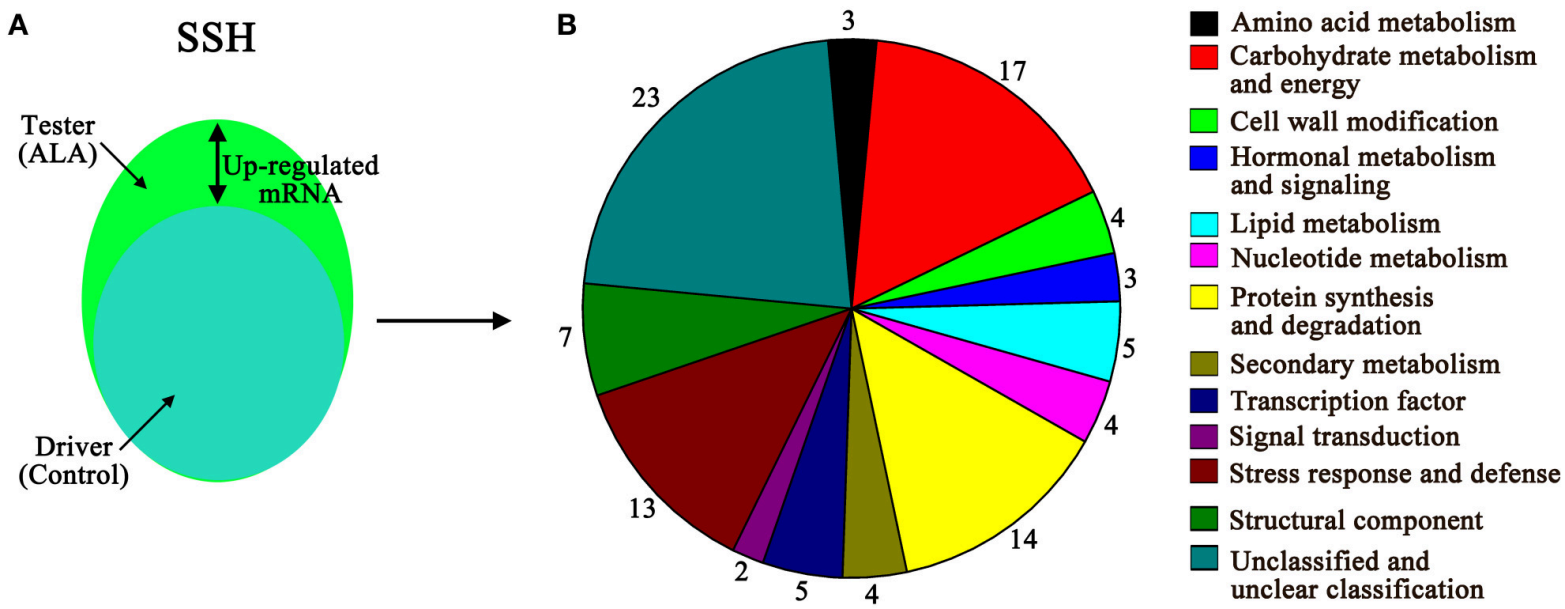
**Up-regulated genes in response to ALA treatment were identified by SSH in apple skin. (A)** Visual representation of the experimental plot of suppression subtractive hybridization (SSH). Tester, ALA-treated apple skin; driver, water-treated apple skin (control). **(B)** The function of unigenes from up-regulated genes pool was classified into 13 functional groups.

To further determine how ALA stimulates fruit coloration, a forward SSH library was constructed with mRNA isolated from ALA-treated to water-treated (control) apple peel (Figure 4A). Subtraction was performed between ALA-treated cDNA (tester) and the control cDNA (driver). Positive clones were selected for sequencing, and the vector and other uninformative sequences were removed. A total of 125 ESTs were successfully sequenced and identified in the apple database and then these ESTs were further assembled into 104 unigenes (Supplementary Table S5). The unigenes present in the forward SSH library were classified into 13 major functional groups, using information from various sources (Figure 4B). Among that, 10 out of 12 functional groups (not including unknown category) were regulated at both protein and mRNA levels. Genes involved in structural component and transcription factor were only detected at mRNA levels. In addition, 1-aminocyclopropane-1-carboxylate oxidase 1 (MDP0000195885), abscisic acid stress ripening protein (MDP0000253074), universal stress protein (MDP0000452572), glutamine amidotransferase-like class I superfamily protein (MDP0000668552), and an uncharacterized protein (MDP0000170439), were detected at both mRNA and protein levels.

Thirty-eight unigenes have been reported to be relevant to fruit ripening (Table 2). The largest category, consisting of 9 genes, was associated with stress response and defense, such as major allergen Mal d 1, a universal stress protein, acidic endochitinase, and dehydrin family protein. Eleven genes were predicted to be associated with primary metabolism, including amino acid metabolism, carbohydrate metabolism and energy, lipid metabolism. Four genes were predicted to be associated with protein synthesis and degradation. In secondary metabolism group, two pigments biosynthesis genes (phytoene dehydrogenase and UFGT) and two aroma production gene (α-farnesene synthase and farnesyl pyrophosphate synthase) were identified. Among the differentially expressed genes, five genes associated with hormonal metabolism and signal transduction. Interestingly, a transcription factor *MADS1*, also referred to as *MADS8* and *SEPALLATA1* (Ireland et al., 2013) was identified from the SSH libraries, which accounted for ripening-related genes.

**Table 2:** **Selected fruit ripening-related genes differentially expressed in ALA-treated apple skin**.

**Unigene No**.	**Accession No**.	**Annotation**	**Ripening associated references**
**AMINO ACID METABOLISM**			
UN070	MDP0000621545	Acetolactate synthase	Citrus (Burns et al., 1999)
**CARBOHYDRATE METABOLISM AND ENERGY**			
UN042	MDP0000196182	NADH dehydrogenase [ubiquinone] iron-sulfur protein 6, mitochondrial	Apple (Janssen et al., 2008)
UN062	MDP0000789873	Glycoside hydrolase	Tomato (Kumar et al., 2015)
UN068	MDP0000253390	Phosphoenolpyruvate carboxylase-related kinase 2	Banana (Law and Plaxton, 1997)
UN076	MDP0000164592	NADH dehydrogenase [ubiquinone] iron-sulfur protein 8	Apple (Janssen et al., 2008)
UN088	MDP0000925483	Transaldolase	Kiwifruit (Minas et al., 2012)
UN092	MDP0000267248	6-Phosphofructokinase	Banana (Turner and Plaxton, 2003)
**LIPID METABOLISM**			
UN005	MDP0000251991	Lipid transport superfamily protein	Apple (Janssen et al., 2008)
UN057	MDP0000262512	lipases; hydrolases, acting on ester bonds	Apple (Sunchung et al., 2006)
UN101	MDP0000305778	Acyl-CoA-binding protein 6	Apple (Sunchung et al., 2006)
**CELL WALL MODIFICATION**			
UN013	MDP0000184228	Pectinesterase-like	Apple (Zhang Z. et al., 2015)
UN014	MDP0000236092	COBRA-like protein 10	Tomato (Cao et al., 2012)
UN020	MDP0000127542	β-Galactosidase	Apple (Zhang Z. et al., 2015)
UN055	MDP0000570395	Glucan endo-1,3-β-glucosidase	Apple (Zhang Z. et al., 2015)
UN079	MDP0000130449	Cytochrome P450 monooxygenase	Apple (Zhang Z. et al., 2015)
**HORMONAL METABOLISM AND SIGNALING**			
UN044	MDP0000171041	S-Adenosylmethionine decarboxylase	Peach (Bregoli et al., 2002)
UN054	MDP0000231245	Probable indole-3-acetic acid-amido synthetase GH3.6	Apple (Schaffer et al., 2013)
UN095	MDP0000195885	1-Aminocyclopropane-1-carboxylate oxidase 1	Apple (Shi et al., 2014)
**SECONDARY METABOLISM**			
UN007	MDP0000170162	UDP-glucose: anthocyanidin 3-O-glucosyltransferase	Apricot (D'Ambrosio et al., 2013) Tomato (Kumar et al., 2015)
UN056	MDP0000148978	Phytoene dehydrogenase	Tomato (Kumar et al., 2015)
UN043	MDP0000199152	α-Farnesene synthase	Apple (Ju and Curry, 2000)
UN096	MDP0000198736	Farnesyl pyrophosphate synthase	Apple (Ju and Curry, 2000)
**PROTEIN SYNTHESIS AND DEGRADATION**			
UN028	MDP0000294774	Proteasome subunit β type 2A	Apple (Shi et al., 2014)
UN071	MDP0000263908	40S ribosomal protein S27	Apple (Zhang Z. et al., 2015)
UN082	MDP0000674266	40s ribosomal protein S25	Apple (Zhang Z. et al., 2015)
UN085	MDP0000222113	Ubiquitin-protein ligase 10/12	Tomato (Kumar et al., 2015)
**SIGNAL TRANSDUCTION**			
UN060	MDP0000258968	Probable protein phosphatase 2C 60	Citrus (Wu et al., 2014)
UN089	MDP0000325949	14-3-3 protein family	Apple (Shi et al., 2014)
**STRESS RESPONSE AND DEFENSE**			
UN001	MDP0000864747	Major allergen mal d 1	Apple (Shi et al., 2014)
UN022	MDP0000770493	Dehydrin family	Tomato (Weiss and Egea-Cortines, 2009)
UN027	MDP0000940313	Acidic endochitinase	Banana (Liu et al., 2012)
UN037	MDP0000452572	Universal stress protein A-like protein	Apple (Shi et al., 2014)
UN039	MDP0000942516	Major allergen mal d 1	Apple (Shi et al., 2014)
UN040	MDP0000295542	Major allergen Mal d 1	Apple (Shi et al., 2014)
UN069	MDP0000153123	Metallothionein-like protein	Strawberry (Aguilar et al., 1997)
UN077	MDP0000103627	Major allergen Mal d 1	Apple (Shi et al., 2014)
UN093	MDP0000253074	Abscisic acid stress ripening protein homolog	Apple (Shi et al., 2014)
**TRANSCRIPTION FACTOR**			
UN038	MDP0000366022	MADS1	Apple (Ireland et al., 2013)

### *MdMADS1* expression is positively correlated with anthocyanin biosynthesis-related genes in ALA treatment

**Figure 5:**
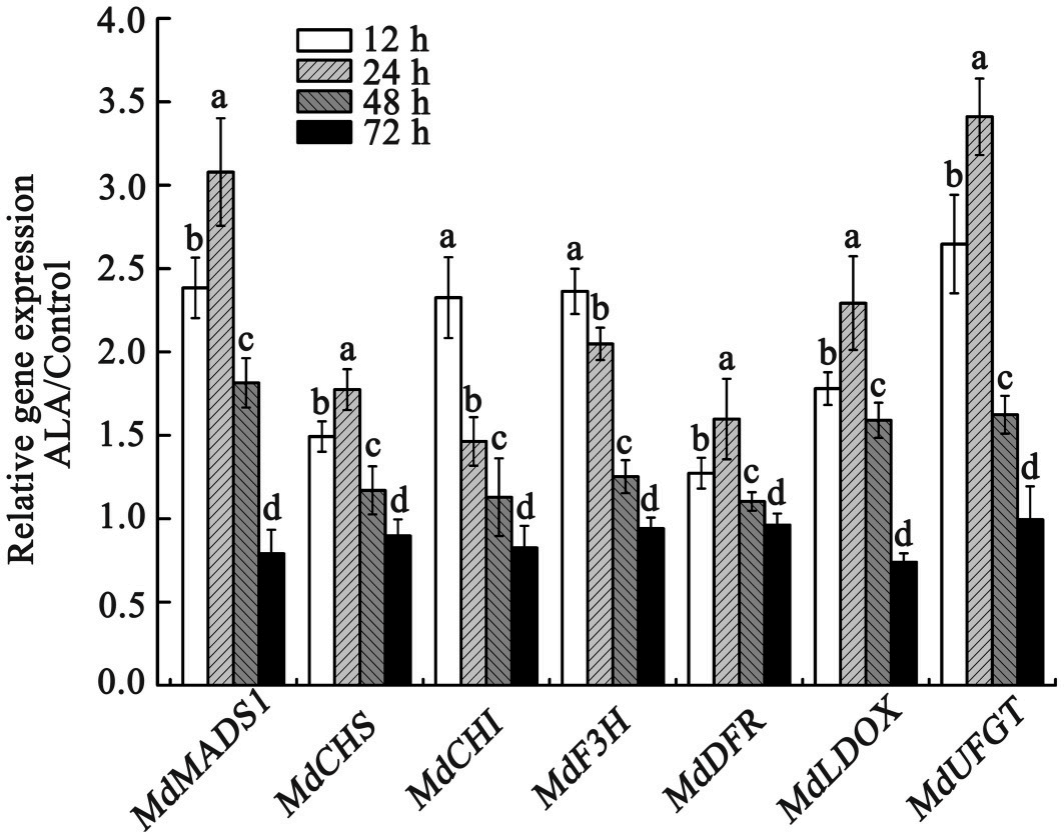
**qRT-PCR analysis of the expression of *MdMADS1* and anthocyanin biosynthetic genes in apple skin under ALA treatment**. The relative expression of *MdMADS1* and anthocyanin biosynthetic genes (*MdCHS, MdCHI, MdF3H, MdDFR, MdLDOX, MdUFGT*) were simultaneously analyzed in apple skin with ALA treatment at 12, 24, 48, and 72 h light duration. The expression level of each gene in control was used as a reference sample at each time point.

The above results indicated that ALA-induced anthocyanin accumulation was probably associated with its regulation of fruit ripening process. Recently, *MdMADS1* was reported to be involved in regulation of apple fruit ripening and *MdMADS1-* antisense lines showed inhibited fruit coloration (Ireland et al., 2013). Therefore, we speculated that the up-regulation of *MdMADS1* expression might play an important role in ALA-induced anthocyanin accumulation. We confirmed by qRT-PCR that ALA indeed enhanced the expression of *MdMADS1* at 12, 24, and 48 h of light irradiation (Figure 5), but decreased it at 72 h. These results indicated that *MdMADS1* was induced by ALA application.

To show the potential role of *MdMADS1* in ALA-induced anthocyanin accumulation, we simultaneously measured the expression of anthocyanin biosynthetic genes (*MdCHS, MdCHI, MdF3H, MdDFR, MdLDOX*, and *MdUFGT*) in response to ALA and investigated the relationship between the expression of *MdMADS1* and anthocyanin biosynthetic genes. We found that the expression of *MdMADS1* showed similar changing pattern to that of anthocyanin biosynthetic genes (*MdCHS, MdDFR, MdLDOX*, and *MdUFGT*) at different light duration (Figure 5). Correlation analysis further showed that the expression of *MdMADS1* was significantly positively correlated with anthocyanin biosynthetic genes, except *MdCHI* and *MdF3H* (Table 3). These results suggested a positive role of *MdMADS1* in ALA-induced improvement of anthocyanin biosynthesis in apple skin.

**Table 3:** **Correlations between the relative expressions of *MdMADS1* and anthocyanin biosynthetic genes under ALA treatment**.

		***MdCHS***	***MdCHI***	***MdF3H***	***MdDFR***	***MdLDOX***	***MdUFGT***
***MdMADS1***	Pearson correlation	0.98^*^	0.62	0.85	0.96^*^	0.99^**^	0.98^*^
	Significance (2-tailed)	0.02	0.38	0.15	0.04	0.00	0.02
	N	4	4	4	4	4	4

* Correlation is significant at the 0.05 level (2-tailed).

** Correlation is significant at the 0.01 level (2-tailed).

### *MdMADS1* is involved in anthocyanin regulation in apple calli

**Figure 6:**
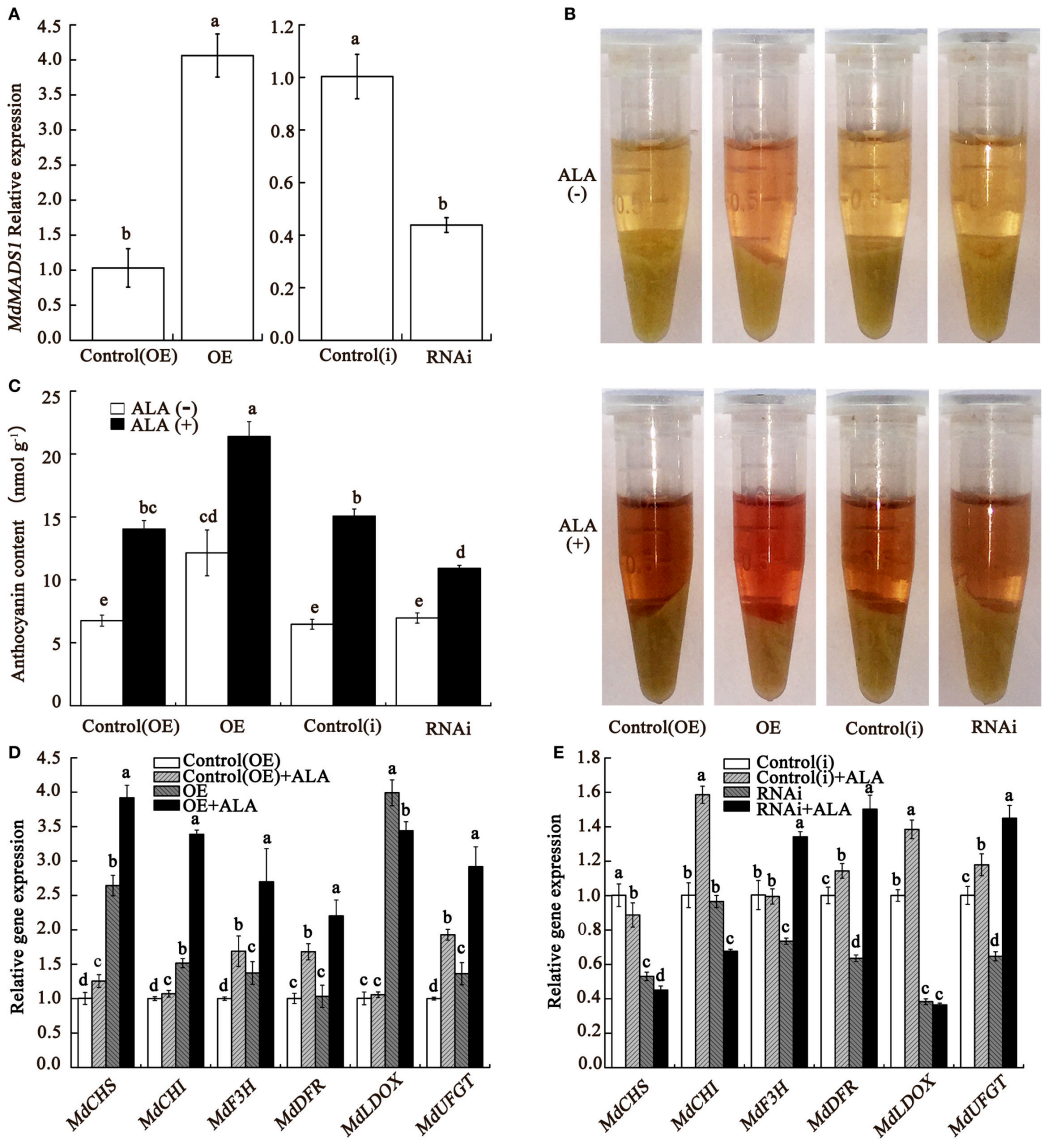
***MdMADS1* involves in ALA-induced anthocyanin accumulation**. The calli were induced from Fuji fruit on MS medium containing 1 mg/L BAP and 1 mg/L 2, 4-D at 25°C in the dark. **(A)** qRT-PCR analysis of the relative expression of *MdMADS1* in transgenic calli. **(B,C)** Color **(B)** and anthocyanin content **(C)** in transgenic calli. **(D,E)** ALA regulated the expression of anthocyanin biosynthetic genes in overexpression **(D)** and RNAi **(E)** transgenic calli. The expression level of each gene in the control(OE) or the control(i) calli was used as a reference sample. The expression level of each gene in calli was used as a reference sample. OE, calli infiltrated with the plasmid for overexpressing *MdMADS1*; Control(OE), calli infiltrated with an empty pBI121 vector; RNAi, calli infiltrated with the plasmid for silencing *MdMADS1*; Control(i), calli infiltrated with an empty pHELLSGATE2 vector.

Callus was a reliable material for anthocyanin-related research (Lalusin et al., 2006; Xie et al., 2012; An et al., 2015). To confirm the role of *MdMADS1* in ALA-induced anthocyanin accumulation, the gene was overexpressed and silenced in apple fruit calli, respectively. In overexpression lines (OE), the expression of *MdMADS1* was significantly increased compared with the corresponding empty plasmids control [control(OE)], and in the silenced lines (RNAi), the expression was drastically reduced compared with the corresponding empty plasmids control [control(i)] (Figure 6A). Under light condition, the appearance revealed that OE calli looked redder in color than the control and RNAi calli (Figure 6B), suggesting that *MdMADS1* overexpression increased anthocyanin accumulation. Spectrophotometric analysis further confirmed that OE calli contained significantly higher content of anthocyanin than the control and RNAi calli (Figure 6C), suggesting that *MdMADS1* play a positive role in anthocyanin accumulation. The expression of the anthocyanin biosynthetic genes were significantly up-regulated and down-regulated in OE and RNAi calli than the control (Figures 6D,E), respectively, indicating that MdMADS1 regulates anthocyanin accumulation by modulating expression of anthocyanin biosynthesis-related genes.

To determine whether *MdMADS1* functioned in ALA-induced anthocyanin accumulation, we compared the anthocyanin content in transgenic calli which were treated with or without ALA. In Figure 6B, results showed that OE calli looked redder and accumulated the markedly higher level of anthocyanin after ALA treatment; ALA-treated RNAi calli were less red than ALA-treated control but much redder than the control without ALA. Spectrophotometric assay showed that ALA significantly promoted the anthocyanin accumulation in OE and RNAi calli (Figure 6C). In addition, we found that the expression levels of anthocyanin biosynthetic genes were higher in OE calli with ALA treatment than that in control(OE) calli with ALA treatment and OE calli, except *MdLDOX* (Figure 6D). Interestingly, ALA reversed the expression levels of three anthocyanin biosynthetic genes (*MdF3H, MdLDOX*, and *MdUFGT*) in RNAi transgenic calli (Figure 6E). These results indicated that *MdMADS1* was involved in ALA-induced anthocyanin accumulation, but the latter was not completely dependent on the former.

## Discussion

ALA has shown to be effective in promoting fruit coloration, yield and quality, so that it has great application potential in horticulture. However, little information is available on regulatory mechanisms behind ALA-induced fruit coloration. Here, two proteomic techniques and SSH were employed to identify the early responses of apple skin coloration to ALA treatment at protein and mRNA levels.

The difference in pretreatment, depth of proteome coverage, analyses of isoforms and quantification statistical power often result in poor correspondence between the proteins identified by different proteomic techniques (Scherp et al., 2011). For example, Yin et al. (2014) found only 9 of 115 in soybean root tips were commonly detected by gel-based and gel-free proteomics under flooding stress. Majeran et al. (2005) reported that among the 125 chloroplast proteins quantified in the three methods (2-DE, ICAT, and label-free), only 20 proteins were quantified in common. Moreover, the SDS-PAGE-based and gel-free-based proteomic techniques were combined to explore the molecular mechanism responsible for low silk production, a total of 17 of 174 changed proteins were common between the two techniques (Wang et al., 2014). Similarly, here, we identified 85 changed proteins using gel-based and gel-free techniques, among which only 12 proteins were commonly identified by two methods. Although most of the proteins were not identified simultaneously by different proteomic techniques, they showed similar altered trends in biological function (Figure 3A, Yin et al., 2014). Our data together with the above previous studies indicate that utilization of different proteomic approaches can lead to a more comprehensive proteome profiling, providing complementary information and hence a better understanding of the mechanisms.

Only five ALA-responsive genes were identified at both protein and mRNA levels, indicating that the expression levels of individual proteins were not strictly correlated with the up-regulated transcripts. Similar results have been reported in several previous studies (Lan et al., 2012; Arcondéguy et al., 2013; Wang et al., 2014). The regulation of mRNA synthesis, the post-transcriptional regulation of mRNA splicing and supervision mechanism, and the post-translational modification of the mRNA translated product can all result in altered protein levels (Arcondéguy et al., 2013). In addition, the different expression time course between mRNA and proteins also can lead to the divergence of mRNA and protein expression levels (Lan et al., 2012; Wang et al., 2014). Although the differentially changed proteins and transcripts did not correspond well at an individual level, the pathway analysis showed that proteome and transcriptome in the present study were well-matched. A total of 10 out of 12 transcriptome-involved pathways were found in the proteome-involved pathways, suggesting that the changed direction of proteins and transcripts were coordinated. Therefore, the proteomics and SSH techniques were mutually complementary and verified in this study, and provide valuable information on mechanisms involved in ALA-regulated apple skin.

Higher levels of anthocyanins are closely related to higher expression of anthocyanin biosynthetic genes. Previous studies have reported that exogenous ALA up-regulated the expression level of key genes in the pathway of anthocyanin biosynthesis (Xie et al., 2013; Feng et al., 2015). At protein level, a 4-coumarate-CoA ligase (No. 54 in Table 1), which catalyses the last step of the phenylpropanoid pathway leading to either lignins or flavonoids (Dixon et al., 2002), was induced by ALA treatment. UFGT is the last enzyme in anthocyanin biosynthetic pathway and has widely been considered as the key enzyme determining apple coloration (Li et al., 2002; Kim et al., 2003). Ju et al. (1995) found that anthocyanin biosynthesis in apple peel was most strongly correlated with UFGT activity. Here, the expression levels of UFGT (No. 50 in Table 1 and UN007 in Table 2) were up-regulated at both transcript and protein level under ALA treatment. These results indicate that up-regulation of biosynthetic genes contributes greatly to ALA-promoted anthocyanin accumulation. Except up-regulation of anthocyanin biosynthetic genes, ALA also repressed cinnamoyl-CoA reductase (No. 52 in Table 1) at protein level in the lignin biosynthetic pathway. Ludwig et al. (2013) reported that there was a competition between lignin and anthocyanin biosynthetic pathways for their common precursors. These results indicate that ALA helps divert the metabolic flux from lignin to anthocyanin pathway, which also contribute to the anthocyanin accumulation. Our results add new evidence supporting the positive regulation of ALA on anthocyanin accumulation.

ALA, as a potential plant growth regulator, is known to be effective in improving plant tolerance to various stresses (Akram and Ashraf, 2013). In this study, ALA alters the expression of numerous genes at mRNA and protein levels associated with various biological processes in apple skin. One of the outstanding abundant classes is protein and genes involved in stress response, indicating the positive role of ALA in defense response of apple skin to stresses. Anthocyanin itself, is a secondary metabolite which shows antioxidant activity (Alessio et al., 2011), and plays essential roles in ameliorating environmental stresses, such as UV-B radiation, drought, and cold temperatures (Chalker-Scott, 1999). In the red-fleshed apples, a recent study suggested that anthocyanin was both associated with the red coloration and the stress tolerance (Wang et al., 2015). Here, the effects of ALA on apple skin included anthocyanin accumulation as well as a stress response. Thus, it is reasonable to speculate that ALA might promote anthocyanins accumulation, participating in stress resistance of apple skin.

**Figure 7:**
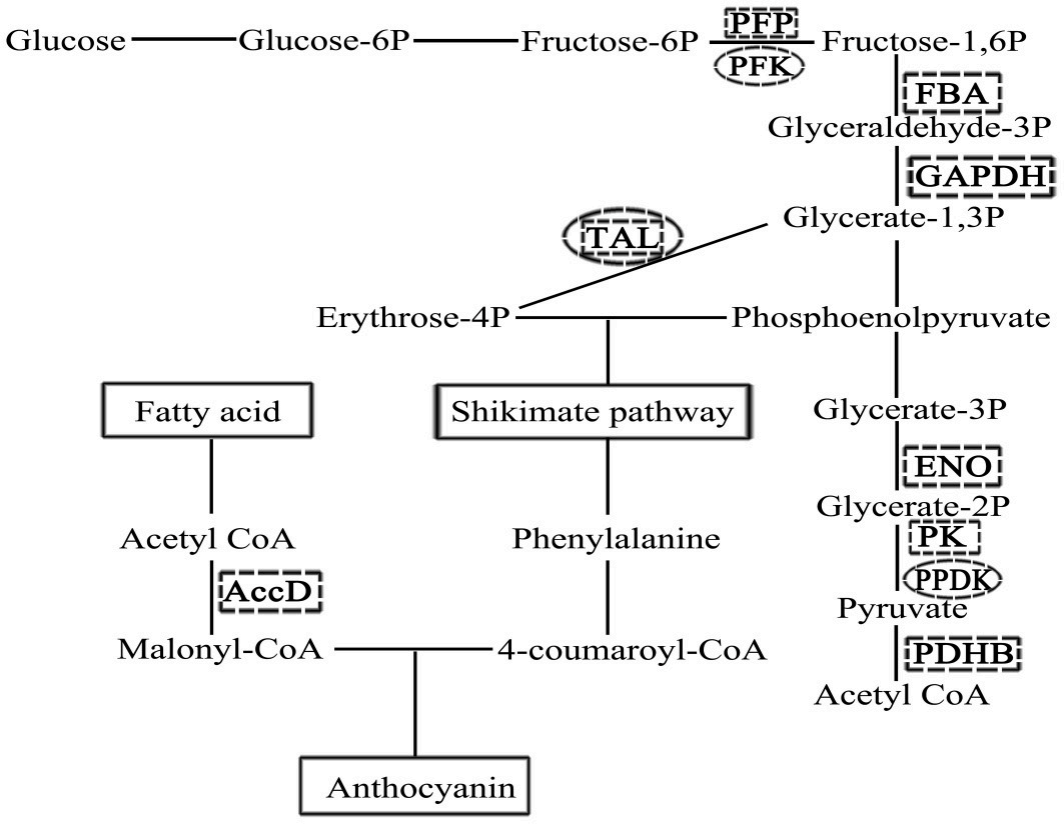
**Schematic representation of the supply of precursors for the anthocyanin biosynthetic pathway**. The formation of two reaction substrates in anthocyanin biosynthesis, malonyl-CoA and 4-coumaroyl-CoA, were linked to glycolysis, pentose phosphate pathway, shikimate pathway, and fatty acid metabolism. Enzymes identified by proteomics (marked with a dotted square) or SSH (marked with a dotted circle) were shown in bold uppercase letters (AccD, acetyl-CoA carboxyltransferase, β subunit; PFP, pyrophosphate: fructose 6-phosphate 1- phosphotransferase; PFK, 6-phosphofructokinase; FBA, fructose-bisphosphate aldolase; GAPDH, glyceraldehyde-3-phosphate dehydrogenase; ENO, enolase; PK: pyruvate kinase; PPDK: pyruvate phosphate dikinase; PDHB, pyruvate dehydrogenase E1 subunit β; TAL: transaldolase).

Another more abundant functional class in ALA treatment is related to primary metabolites involved in the metabolism of amino acid, sugar, and fatty acid. Li et al. (2014) found that the differences between anthocyanin concentrations in the pear peel of green “Anjou” and its bud mutation “Red Anjou” were accompanied with up-regulated of sorbitol metabolism and altered amino acid metabolism in the peel of “Red Anjou.” It is demonstrated that the manipulation of primary metabolism can change the production of secondary metabolites (Henkes et al., 2001; Dauwe et al., 2007). In this study, change of primary metabolism in apple skin under ALA treatment indicates that they may be crucial at the initial stage of ALA-induced anthocyanin accumulation. Therefore, we attempted to capture the potential links between primary metabolism and anthocyanin accumulation in ALA treatment (Figure 7). The first key step of the anthocyanin biosynthesis, catalyzed by chalcone synthase, involves two reaction substrates malonyl-CoA and 4-coumaroyl-CoA (Winkel-Shirley, 2001). In plants, the formation of malonyl-CoA from the carboxylation of acetyl-CoA in fatty acid chain elongation pathway is catalyzed by acetyl-CoA carboxylase, which is an ATP-dependent biotinylated protein complex (Sasaki and Nagano, 2014). The β subunit of this protein (No. 36 in Table 1) was identified here, and it appears positively regulated by ALA. Another substrate 4-coumaroyl-CoA is converted by phenylalanine. The formation of phenylalanine in shikimate pathway is connected to two intermediate metabolites, phosphoenolpyruvate of glycolysis pathway and erythrose 4-phosphate of the pentose phosphate pathway in carbohydrate metabolism (Tzin and Galili, 2010). Here, we found that genes related to glycolysis, such as pyrophosphate: fructose 6-phosphate 1-phosphotransferase (No. 24), glyceraldehyde-3-phosphate dehydrogenase (No. 16 and 27), enolase (No. 20), fructose-bisphosphate aldolase (No. 19), pyruvate kinase (No. 29), and pyruvate dehydrogenase E1 subunit β (No. 12) were regulated and at protein level (Table 1) while 6-phosphofructokinase (UN092) and pyruvate phosphate dikinase (UN016) was up-regulated at mRNA level (Table 2) under ALA treatment. Moverover, plastidial and cytosolic enolase, respectively, have specific functions in metabolism (Voll et al., 2009). In the present study, the subcelluar locations of enolase was predicted using an online tool Plant-mPLoc (http://www.csbio.sjtu.edu.cn/bioinf/plant/. Chou and Shen, 2010), suggesting that glycolytic enzyme is a cytosolic protein. This data supported the suggestion that, cytosolic enolase plays a central role to modulate the synthesis of aromatic amino acids and secondary phenylpropanoid compounds, even in the absence of a complete glycolysis pathway in the plastids (Voll et al., 2009; Eremina et al., 2015; Negri et al., 2015). Therefore, our results suggest that carbon flux into anthocyanin biosynthesis is activated by ALA during fruit coloration period. Transaldolase links the pentose phosphate pathway to glycosis and it was up-regulated by ALA at both protein and mRNA levels (No. 10, 13, 26 in Table 1 and UN088 in Table 2). The high expression level of this gene could result in an accumulation of erythrose 4-phosphate. These results together indicate that the promotion of ALA-induced anthocyanin synthesis may also be associated with an ALA-enhanced supply of precursors from primary metabolism to secondary metabolism.

It is well-documented that the expression of genes related to stress responses, primary and secondary metabolism, cell wall metabolism, and hormonal metabolism contribute to fruit ripening process (Shi et al., 2014; Kumar et al., 2015). Previous studies have suggested that ALA enhanced fruit maturation with a promotion of many ripening-related biological events (Watanabe et al., 2006). However, to date, there is little evidence elucidating the molecular mechanism how ALA regulates fruit ripening. In this study, about 85% of changed proteins and 38% of up-regulated mRNA in ALA-treated apple skin have a secondary function associated with fruit ripening (Figure 3C and Table 2). This result is not surprising because most fruits accumulate anthocyanin only in their ripening phase (Jaakola et al., 2002; Whale and Singh, 2007; Negri et al., 2008; Bureau et al., 2009). Our results demonstrate that ALA regulates the expression of ripening-related genes and therefore provide more evidence on the regulation of fruit ripening by ALA.

Our results suggest a role of ALA in the modulation of apple skin physiology by regulating fruit ripening-related processes. Transcription factors play important roles in controlling the switch to the ripening phase in fruits (Giovannoni, 2007; Karlova et al., 2014). Since studies on *ripening inhibitor* (*Rin*) mutation in tomato revealed SEPALLATA (SEP) subfamily of MADS-box genes play a key role in the developmental control of fruit ripening (Vrebalov et al., 2002), there is evidence that members of *SEP*-*like* orthologous gene families are involved in fruit development in other species, such as strawberry (Seymour et al., 2011), and banana (Elitzur et al., 2010). In fact, some MADS box TF from the AP1/SQUA class have been implicated in controlling of anthocyanin biosynthesis. *IbMADS10* expression was correlated with red pigmentation in sweet potato, and ectopic expression resulted in anthocyanin accumulation in transgenic sweet potato calli and transgenic *Arabidopsis* (Lalusin et al., 2006). *VmTDR4* expression was linked with color development and anthocyanin-related gene expression in bilberry (*Vaccinium myrtillus*), while silencing of this gene reduced anthocyanin levels (Jaakola et al., 2010). Meanwhile, *SEP*-*like* genes have also been reported to be associated with the accumulation of anthocyanin in pear (Wu et al., 2013). In strawberry, suppression of *SEP1/2*-*like FaMADS9* resulted in delayed ripening with respect to anthocyanin accumulation. Therefore, *SEP-like* gene regulated fruit ripening process including skin color change. Here, we identified an ALA-responsive gene, *MdMADS1* (UN038 in Table 2), which belongs to the SEP subgroup (Sung and An, 1997). In apple, fruits of the *MdMADS1* antisense lines do not ripen in terms of both developmentally controlled ripening characters, such as starch degradation and background color change of fruit skin (Ireland et al., 2013). However, the authors did not investigate anthocyanin biosynthesis and accumulation in the transgenic fruits, and the exact relationship between *MdMADS1* and fruit coloration remains unknown. Our SSH result showed that ALA up-regulated the expression of *MdMADS1*, and this regulation was further confirmed by real-time quantitative PCR. Furthermore, under ALA treatment, the expression of *MdMADS1* was significantly positively correlated with that of anthocyanin biosynthetic genes, including *MdCHS, MdDFR, MdLDOX*, and *MdUFGT*. These results indicate that *MdMADS1* may play an important role in ALA-promoted anthocyanin accumulation. To prove the role of *MdMADS1*, we transformed and successfully obtained transgenic fruit calli. Overexpression of *MdMADS1* led to more anthocyanin accumulation. The six anthocyanin biosynthetic genes were up-regulated in overexpression transgenic calli and down-regulated in RNAi transgenic calli. Anthocyanin accumulated in overexpression and RNAi transgenic calli treated by ALA was higher not only than their corresponding control but also than that in transgenic calli without ALA. These results indicate that *MdMADS1* involves in ALA-induced anthocyanin accumulation, but the latter is not completely dependent on the former.

In apple fruit, *MdMADS1* is a master regulator that controls fruit ripening process including the initiation of ethylene biosynthesis (Ireland et al., 2013; Schaffer et al., 2013). The central role of ethylene in apple fruit ripening has been well-studies (Dandekar et al., 2004; Schaffer et al., 2007). Of these, ethylene appeared to be a key factor regulating anthocyanin biosynthesis and color development in apple fruit (Whale and Singh, 2007). In the present study, we also identified 1-aminocyclopropane-1-carboxylate oxidase 1 (ACO1, No. 34 in Table 1 and UN095 in Table 2) at protein and mRNA levels, a key enzyme in ethylene biosynthesis, and ethylene receptor 2 (ERS2, No. 35 in Table 1) at protein level, an ethylene signal transduction gene, suggesting that *MdMADS1* might regulate ALA-induced anthocyanin accumulation by its regulation of ethylene metabolism and action. However, the role of ethylene in apple cultivar “Fuji” fruit may be quiet limited, because the ethylene production in “Fuji” fruit was significant lower than that in other cultivars (Harada et al., 2000; Tatsuki et al., 2007). In addition, the skin color change in *MdMADS1* antisense fruits was not completely compensated for by exogenous ethylene (Ireland et al., 2013), suggesting a non-ethylene-dependent pathway exists in MdMADS1-regulated fruit coloration. In this study, the function of *MdMADS1* was tested in fruit calli, proving that the regulation of *MdMADS1* on anthocyanin biosynthesis is at least partly independent of fruit ripening process. Our data firstly reveal a positive regulation role of *MdMADS1* in anthocyanin biosynthesis. Further study is needed to reveal the mechanisms behind ripening-related and non-ripening-related anthocyanin accumulation mediated by *MdMADS1*. Except *MdMADS1*, ALA promotes anthocyanin accumulation has been linked to regulatory genes *MYB, bHLH*, and *WD40* (Xie et al., 2013). Therefore, the interaction between the MdMADS1 and MYB–bHLH–WD40 complexes need further study.

## Conclusion

In summary, the integrated proteomics and SSH techniques, in this study, provide us a comprehensive understanding of biological events that are relevant to ALA-improved fruit coloration. Based on the results, we identified a positive regulator, *MdMADS1*, and verified its role in ALA-induced anthocyanin accumulation by further functional characterization. In apple fruits, the expression of *MdMADS1* was induced by ALA, which was significantly positively correlated to that of anthocyanin biosynthetic genes under ALA treatment. In fruit calli, overexpressed *MdMADS1* enhanced anthocyanin content, and the accumulation was further enhanced by ALA treatment. However, silenced *MdMADS*1 cannot completely repress anthocyanin accumulation in ALA-treated calli. The results indicate synergistic or additive responses between ALA and *MdMADS1* exists for regulation of apple skin anthocyanin accumulation. In addition, verification in apple calli, a non-fruit test system, suggested the regulation of *MdMADS1* on anthocyanin biosynthesis is partially independent of fruit ripening process. Our results contribute to the understanding of ALA-stimulated fruit coloration and expand the existing information on transcription regulation of anthocyanin accumulation in fruit. Further study is needed to elucidate how MdMADS1 regulates the genes encoding anthocyanin biosynthetic pathway enzymes.

## Author contributions

XF, YA conceived and designed research. XF, JZ, MS carried out all the experiments. XF, YA analyzed the data. YA, LW wrote the manuscript. All authors read and approved the manuscript.

## Funding

This research was supported by the Natural Science Foundation of Jiangsu Province, China (BK20140702), the National Natural Science Foundation of China (31401820), and the Fundamental Research Funds for the Central Universities (KJQN201538). The funders had no role in study design, data collection and analysis, decision to publish, or preparation of the manuscript.

### Conflict of interest statement

The authors declare that the research was conducted in the absence of any commercial or financial relationships that could be construed as a potential conflict of interest.
