# Supplementary material for: Proteomics and SSH Analyses of ALA-Promoted Fruit Coloration and Evidence for the Involvement of a MADS-Box Gene, MdMADS1
Source: Front Plant Sci. 2016 Nov 7;7:1615. doi: 10.3389/fpls.2016.01615 (PMC5098116; doi:10.3389/fpls.2016.01615)
Supplement: Supplementary file 1 [file Table1.DOC]

**Table S1** Primers used in this study

| MdMAD1(OE)-F | GAGAACACGGGGGACATGGGGAGAGGAAGAGTGGAG (homologous to the vector underlined) | forward primer for overexpressing *MdMADS1* |
| --- | --- | --- |
| MdMAD1(OE)-R | CGATCGGGGAAATTCTCAAAGCATCCATCCAGGGATG | reverse primer for overexpressing *MdMADS1* |
| MdMAD1(i)-F | GGGGACAAGTTTGTACAAAAAAGCAGGCTCAATCCCAAGGATTCTTCCA | forward primer for silencing *MdMADS1* |
| MdMAD1(i)-R | GGGGACCACTTTGTACAAGAAAGCTGGGTGCATAATTGGGCATGCTTTC | reverse primer for silencing *MdMADS1* |
| MdMAD1-F | TGCTGGACCAACTTTCTG | forward qRT-PCR primer for *MdMADS1* |
| MdMAD1-R | TCACTTGCTGGGCATTAG | reverse qRT-PCR primer for *MdMADS1* |
| MdCHS-F | GGAGACAACTGGAGAAGGACTGGAA | forward qRT-PCR primer for *MdCHS* |
| MdCHS-R | CGACATTGATACTGGTGTCTTCA | reverse qRT-PCR primer for *MdCHS* |
| MdCHI-F | GGGATAACCTCGCGGCCAAA | forward qRT-PCR primer for *MdCHI* |
| MdCHI-R | GCATCCATGCCGGAAGCTACAA | reverse qRT-PCR primer for *MdCHI* |
| MdF3H-F | TGGAAGCTTGTGAGGACTGGGGT | forward qRT-PCR primer for *MdF3H* |
| MdF3H-R | CTCCTCCGATGGCAAATCAAAGA | reverse qRT-PCR primer for *MdF3H* |
| MdDFR-F | GATAGGGTTTGAGTTCAAGTA | forward qRT-PCR primer for *MdDFR* |
| MdDFR-R | CGGCCGGAATCGGAATCAATC | reverse qRT-PCR primer for *MdDFR* |
| MdLDOX-F | CCAAGTGAAGCGGGTTGTGCT | forward qRT-PCR primer for *MdLDOX* |
| MdLDOX-R | CAAAGCAGGCGGACAGGAGTAGC | reverse qRT-PCR primer for *MdLDOX* |
| MdUFGT-F | CCACCGCCCTTCCAAACACTCT | forward qRT-PCR primer for *MdUFGT* |
| MdUFGT-R | CACCCTTATGTTACGCGGCATGT | reverse qRT-PCR primer for *MdUFGT* |
| MdActin-F | TGACCGAATGAGCAAGGAAATTACT | forward qRT-PCR primer for *MdActin* |
| MdActin-R | TACTCAGCTTTGGCAATCCACATC | reverse qRT-PCR primer for *MdActin* |
