# Supplementary material for: Proteomics and SSH Analyses of ALA-Promoted Fruit Coloration and Evidence for the Involvement of a MADS-Box Gene, MdMADS1
Source: Front Plant Sci. 2016 Nov 7;7:1615. doi: 10.3389/fpls.2016.01615 (PMC5098116; doi:10.3389/fpls.2016.01615)
Supplement: Supplementary file 2 [file Table2.DOC]

**Table S2 MS results for apple skin proteins with significant abundance changes in ALA treatment *vs*. the control**

| Spot No. | Accession No. | Annotation | Ratio ^a^ | *P*-value | Mascot Score | Matched % Cov. | Matched peptides | Protein MW/PI | Sequence (Ions score) |
| --- | --- | --- | --- | --- | --- | --- | --- | --- | --- |
| 1 | MDP0000246775 | Thaumatin-like protein 1a | 1.70 | 0.0297 | 188 | 19 | 4 | 26.819/4.97 | ASQSVDAPSPWSGR (90)  ASQSVNAPSPWSGR (24)  SACLAFGDSK (45)YCCTPPNNTPETCPPTEYSEIFEK (110) |
| 2 | MDP0000170439 | Uncharacterized protein | 0.47 | 0.0324 | 695 | 23 | 9 | 54.837/4.78 | KPEYGQSGSEYGSGYQR (166)  RPEADEPSSEYTGYGR (93)  KPEHEEPESEYGSGHGR (143)  APEPEYGSGYGR (100)  KPEFEAPPAEFGSGYGR (143)  RTGYGDEPPPPR (81)  MTGYGDEPPPPR (82)  TGYGDEPPPPR (92)  SEEQKYGEEGYGR (83) |
| 3 | MDP0000170439 | Uncharacterized protein | 0.51 | 0.0132 | 619 | 21 | 8 | 54.837/4.78 | KPEYGQSGSEYGSGYQR (150)  RPEADEPSSEYTGYGR (96)  APEPEYGSGYGR (91)  KPEYEAPESEYGSGYGR (124)  KPEFEAPPAEFGSGYGR (153)  RTGYGDEPPPPR (77)  TGYGDEPPPPR (83)  SEEQKYGEEGYGR (83) |
| 4 | MDP0000121897 | Adenine phosphoribosyltransferase | 2.38 | 0.0266 | 597 | 76 | 6 | 12.992/5.03 | GFIFGPPIALAIGAK (91)  VISEEYSLEYGTDIMEMHVGAVEAGER (212)  VISEEYSLEYGTDIMEMHVGAVEAGER (83)  ALIIDDLIATGGTLGAAIK (146)  VGVHVVECACVIELPELK (126)  LGEKPLFVLVSGDA (113) |
| 5 | MDP0000663130 | Acireductone dioxygenase | 0.40 | 0.0063 | 252 | 7 | 4 | 63.504/6.5 | LKNFFEEHLHTDEEIR (104)  NFFEEHLHTDEEIR (120)  YAVDGSGYFDVR (103)  GGMIVLPAGIYHR (34) |
| 6 | MDP0000234480 | Transaldolase | 2.22 | 0.0375 | 769 | 37 | 10 | 48.253/6.38 | EGQSPWYDNLCRPVTDLLPLIASGVR (55)  AISTSNAYNDQFR (112)  DIESAYWELVVK (72)  LFEAIYDQTDAGDGYVSVEVSPR (179)  YEEVIDAYLDGLEASGLSDLSR (186)  VTSVASFFVSR (75)  IGNPEALDLR (83)  AAVAQAALAYQLYQK (106)  LGIDWSYVGNQLELEGVDSFKK (143)  SFDSLLDTLQEK (85) |
| 7 | MDP0000333058 | Acetyl-CoA carboxyltransferase beta subunit | 2.27 | 0.0436 | 374 | 28 | 4 | 26.854/4.46 | SNYSNVDHLVGDR (92)  NFISDDTFFVR (86)  DSYSIYFDIENTNFEIDNNR (186)  SSFYSYQTSSYMNNAPQSDDTR (122) |
| 8 | MDP0000298502 | Heat shock protein 70 | 2.03 | 0.0156 | 795 | 14 | 10 | 96.066/5.42 | QFAAEEISAQVLR (111)  AVVTVPAYFNDSQR (106)  IINEPTAASLAYGFDR (122)  AKFEELCSDLLDR (80)  TPVENSLR (38)  LAWKDIDEVILVGGSTR (89)  DIDEVILVGGSTR (120)  IPAVQELVR (81)  SEVFSTAADGQTSVEINVLQGER (208)  KQDITITGASTLPSDEVER (185) |
| 9 | MDP0000416706 | Heat shock protein 70 | 1.68 | 0.0001 | 830 | 18 | 10 | 78.735/5.01 | QAVVNPENTFFSVK (50)  QFAAEEISAQVLR (115)  AVVTVPAYFNDSQR (106)  IINEPTAASLAYGFDR (125)  AKFEELCSDLLDR (92)  LAWKDIDEVILVGGSTR (111)  DIDEVILVGGSTR (115)  IPAVQELVR (74)  SEVFSTAADGQTSVEINVLQGER (215)  KQDITITGASTLPSDEVER (159) |
| 10 | MDP0000416706 | Heat shock protein 70 | 1.80 | 0.0416 | 788 | 18 | 10 | 78.735/5.01 | QAVVNPENTFFSVK (13)  QFAAEEISAQVLR (109)  IINEPTAASLAYGFDR (134)  AKFEELCSDLLDR (85)  LAWKDIDEVILVGGSTR (109)  DIDEVILVGGSTR (115)  IPAVQELVR (70)  SEVFSTAADGQTSVEINVLQGER (210)  LDGIPPAPR (63)  KQDITITGASTLPSDEVER (186) |
| 11 | MDP0000147610 | F-type ATPases | 2.11 | 0.0037 | 209 | 26 | 6 | 19.769/5.12 | NIDWEGMAK (51)  NIDWEGMAK (30)  LLVSDEAR (21)  FSQEPEPIDWEYYR (108)  FSQEPEPIDWEYYRK (75)  YVDTVTPQYKPK (74) |
| 12 | MDP0000201559 | Plasma membrane-associated cation-binding protein 1-like isoform 2 | 0.64 | 0.0175 | 252 | 24 | 2 | 23.495/5.16 | VSTFIVTEETVEPLPPPAEATK (90)  KPKPTEPEAAAEAPPPKVEESAAAEPPKP (191) |
| 13 | MDP0000270640 | 14-3-3 protein 7-like | 1.96 | 0.0064 | 321 | 30 | 6 | 30.545/5.31 | VAKLDVELSVEER (55)  LDVELSVEER (80)  ILSSIEQKEEAK (50)  ICHDILTVVDYHLLPSSSTGESTVFYHK (134)  MKGDYYR (17)  AYLTAVDTAASELPPTHPIR (144) |
| 14 | MDP0000195885 | 1-aminocyclopropane-1-carboxylate oxidase 1 | 1.84 | 0.0096 | 269 | 17 | 4 | 35.559/5.24 | ATFPVVDLSLVNGEER (135)  GLDDVQSEIHDLDWESTFFLR (155)  VIAQSDGTR (36)  FVFDDYMK (50) |
| 15 | MDP0000225318 | Adenosine kinase | 0.30 | 0.0321 | 461 | 31 | 6 | 32.957/5.28 | LAGVNVQYYEDDKAPTGTCAVCVVGGER(205)  SLVANLSAANCYK (67)  TEHLKQPENWALVEK (70)  ALPYMDYVFGNETEAR (96)  ALPYMDYVFGNETEAR (87)  ITVITQGADPVVVAEDGKVK (109) |
| 16 | MDP0000769597 | Pyruvate dehydrogenase E1 subunit beta | 1.57 | 0.0269 | 291 | 13 | 6 | 46.853/6.62 | VFLMGEEVGEYQGAYK (58)  SNYMSAGQINVPIVFR (71)  SNYMSAGQINVPIVFR (58)  IAGADVPMPYAANLER (137)  IAGADVPMPYAANLER (42)  LAVPQVEDIVR (75) |
| 17 | MDP0000316685 | Transaldolase | 1.87 | 0.0024 | 644 | 21 | 9 | 74.179/8.89 | EGQSPWYDNLCRPVTDLLPLIASGVR (59)  AISTSNAYNDQFR (94)  DIESAYWELVVK (72)  LFESIYDQTDAGDGYVSVEVSPR (188)  YEAVIDAYLDGLEASGLSDLSR (190)  VTSVASFFVSR (83)  IGTPEALDLR (45)  TIDSNVSEAEGIYSALEK (114)  SFDSLLDTLQEK(82) |
| 18 | MDP0000303430 | Heat shock protein 90 | 2.41 | 0.0313 | 490 | 15 | 10 | 81.374/5.08 | LDAQPELFIR (61)  ADLVNNLGTIAR (83)  ITLFLKEDQLEYLEER (122)  RAPFDLFDTR (59)  APFDLFDTR (51)  GVVDSDDLPLNISR (90)  SGDELTSLKDYVTR (91)  KAVENSPFLER (70)  AVENSPFLER (74)  IVDSPCCLVTGEYGWTANMER (114) |
| 19 | MDP0000277802 | MLP-like protein 329 | 2.33 | 0.0187 | 297 | 44 | 7 | 17.828/5.41 | FISNQHYDFPK (75)  AASDKIHDVAVHEGDWETSGSVK (102)  IHDVAVHEGDWETSGSVK (110)  YTIDGNVETYK (40)  YTIDGNVETYKEK (65)  VEIDEANKR (25)  IIFQVTPNSEGGSVK (76) |
| 20 | MDP0000764262 | 3,4-dihydroxy-2-butanone kinase | 0.32 | 0.0043 | 424 | 16 | 7 | 60.855/5.22 | IAGAAAAAGLSLADVAAEAR (139)  QILSPETNYVPITR (84)  LQLEHGLAVDR (84)  IPVPLPPSR (63)  NYPLNDATETVNEIGSSIR (117)  YGGASAGYR (39)  TMLDALIPASTVLQER (97) |
| 21 | MDP0000248012 | Vacuolar proton pump subunit A | 2.32 | 0.0389 | 695 | 23 | 10 | 69.052/5.43 | R.LTTFEDSEKESEYGYVR (121)  VGHDNLIGEIIR (107)  DTVLELEFQGQK (80)  FTMLQTWPVR (35)  LAADTPLLTGQR (82)  YSNSDTVVYVGCGER (125)  EASIYTGITIAEYFR (143)  YSTALESFYDQFDPDFINIR (162)  EVLQREDDLNEIVQLVGK (72)  NIIHYFNLANQAVER (87) |
| 22 | MDP0000103621 | Major allergen mal d 1 | 2.00 | 0.0357 | 543 | 55 | 7 | 17.716/5.79 | GVCTFENEFTSEIPPSR (116)  GVCTFENEFTSEIPPSR (128)  AFVLDADNLIPK (89)  QAEILEGNGGPGTIK (90)  QAEILEGNGGPGTIKK (94)  HRIDSIDEASYSYSYTLIEGDALTDTIEK (132)  LIESYLKDHPDAYN (97) |
| 23 | MDP0000199034 | L-ascorbate peroxidase | 1.56 | 0.0085 | 517 | 21 | 9 | 49.814/6.47 | NCAPLMLR (50)  TGGPFGTMR (63)  TGGPFGTMR (31)  CPAEQSHGANNGLDIAVR (121)  DAPEPPPEGR (65)  TMGLSDKDIVALSGGHTLGR (143)  ALLDDPVFRPLVEK (86)  YAADEDAFFADYAEAHMR (176)  YAADEDAFFADYAEAHMR (80) |
| 24 | MDP0000248823 | Aascorbate peroxidase 6 | 2.00 | 0.0003 | 336 | 17 | 7 | 57.957/9.6 | LGWHDAGTYNK (62)  NIEEWPQR (40)  VDVSAPEQCPEEGRLPDAGPPSPADHLR (98)  MGLNDKEIVALSGAHTLGR (124)  MGLNDKEIVALSGAHTLGR (94)  DGPGAPGGQSWTAQWLK (87)  FDNSYFK (41) |
| 25 | MDP0000287459 | Aldo/keto reductase | 0.35 | 0.0076 | 436 | 22 | 9 | 49.658/6.2 | NIDDNIGSLR (51)  IEPNQMLVNGSPEYVR (88)  IEPNQMLVNGSPEYVR (57)  LGVDYIDLYYQHR (87)  YIGLSEASPDTIR (101)  YIGLSEASPDTIRR (28)  DIEEEIVPLCR (78)  ELGIGIVPYSPLGR (100)  VVESLPANNFLVSGFCPR (120) |
| 26 | MDP0000300513 | Vacuolar proton ATPase subunit C | 2.86 | 0.0042 | 534 | 29 | 7 | 35.12/5.43 | QIEELER (26)  VSGVESSALTVDGVPVDSYLTR (210)  VAEYNNVR (55)  DWLSSYETLTNYVVPR (149)  LFEDNEYALYTVTLFNR (129)  DFEYSSEAQESR (113)  LVQDQENLR (73) |
| 27 | MDP0000296243 | NAD(P)-binding Rossmann-fold superfamily protein | 0.31 | 0.0002 | 354 | 13 | 8 | 73.97/5.73 | STEAENCEANAVMLR (89)  STEAENCEANAVMLR (30)  HYVGPFELFGK (66)  IQPHEPPFTEDLPR (85)  NEAFNINNGDVFK (90)  VLAEQFGIEEYGIDEGGGR (155)  VWEEIVR (44)  EHGFLGFR (53) |
| 28 | No detected | | | | | | | | |
| 29 | MDP0000806502 | 4-hydroxyphenylpyruvate dioxygenase | 0.55 | 0.0408 | 212 | 9 | 3 | 50.634/5.75 | K.SDLSTGNQTHASYLLR (95)  SGDLNFLFTAPYSPTLTR (118)  AFSATHGLGVR (77) |
| 30 | MDP0000609966 | Polyphenol oxidase | 1.66 | 0.0496 | 217 | 10 | 4 | 67.847/6.23 | LPDRGPLR (17)  WYLYFYEK (37)  DPLFYSHHSNVDR (108)  AAVSSSDLTTTFPATLSNTISVEVTRPSATK (143) |
| 31 | MDP0000416548 | β-Galactosidase | 1.91 | 0.0326 | 321 | 12 | 7 | 81.529/5.69 | YVPGIAFR (37)  TAGGPFMATSYDYDAPLDEYGLPR (111)  LSFSQNVNLR (78)  GQIWINGQSVGR( 61)  HWPGYIAR (40)  THCGEPSQR (37)  SWLTPTGNLLVVFEEWGGDPSR(156) |
| 32 | MDP0000198482 | Glyceraldehyde-3-phosphate dehydrogenase | 1.61 | 0.0340 | 629 | 36 | 8 | 44.97/7.6 | IATTRDDIEVVAVNDPLVDPK(96) YMAYMFK(42) YDSTHGIFDGSISVVDNSTLEINGK(166) RDPAEIPWGDYGVEYVVESSGIFTTLEK(36) VIHEEFGILEGLMTTVHATTATQK(106) VPTPNVSVVDLTCR(112) GILGYTEEDVVSNDFVGDSR(188) LVSWYDNEWGYSNR(99) |
| 33 | MDP0000052862 | UDP-glucose: anthocyanidin 3-O-glucosyltransferase | 1.98 | 0.0289 | 495 | 21 | 9 | 54.081/5.85 | ISHSHPFISFR(64) NISVPAITFDFIR(102) DTVFEFPGWK(56) EIANGLEASGQR(84) GVLPEGFLER(72) NVLVTDMEIAIGVEQR(116) NVLVTDMEIAIGVEQR(93) DEEGGFVSGEEVER(130) LGEMASAALGETGSSTR(81) |
| 34 | MDP0000221498 | Polyphenol oxidase | 2.12 | 0.0295 | 133 | 6 | 4 | 67.847/6.23 | MYLYFYER(36) TPHLFFGHEYR(65) FPATFDSK(15) FDVFINDDAESLSR(88) |
| 35 | MDP0000146975 | Glycyl-tRNA synthetase 1 | 0.57 | 0.0446 | 296 | 15 | 7 | 73.151/5.73 | K.SNVLAFWR(25) LPFAAAQIGQAFR(85) FAEVAELEFLMFPR(39) GIVNNETLGYFIGR(94) SGEALVAHEKYPEPR(49) VFTPSVIEPSFGIGR(100) TDELGVPFAITVDSTSSVTLR(94) |
| 36 | MDP0000868045 | Abscisic acid response protein | 3.31 | 0.0016 | 216 | 26 | 3 | 19.860/8.61 | SHLHNQHGAQPTDAYGNPIQR(171) EHHGVTGALHR(77) SGSSSSSSSEDDGLGGR(15) |
| 37 | MDP0000249227 | Soluble inorganic pyrophosphatase | 1.85 | 0.0268 | 327 | 26 | 7 | 48.022/5.06 | VQPPQNAIEPHVTYHHDHSSHPPLNER(108) RSIAAHPWHDLEIGPGAPK(48) SIAAHPWHDLEIGPGAPK(102) IFNCVIEIPK(44) ILYSSVVYPHNYGFIPR(96) TLCEDNDPLDVLIIMQEPVVPGCFLR(85) IIAVCADDPEYR(28) |
| 38 | MDP0000755275 | Aldose 1-epimerase | 1.54 | 0.0226 | 805 | 45 | 10 | 37.126/5.90 | M.ADANQKPEIFELNNGSMR(131) ADANQKPEIFELNNGSMR(68) LADVVLGFDSVDPYVK(137) GVAPYFGSIVGR(94) QIWEVAELKK(66) YHSHDGEEGYPGNLSLTATYTLTSSTTMR(167) GTPFDFTAEK(49) GTPFDFTAEKR(82) VGESIHEVGLGYDHNYVLDCGEEKEGLK(121) HSGLCLETQGFPNAINTPNFPSIVVQPGER(211) |
| 39 | MDP0000188304 | Aminoacylase-1 | 0.27 | 0.0012 | 406 | 20 | 7 | 45.149/5.92 | CVGIQYLEAIR(54) GLNVGIVLDEGLASPTENYR(165) VPPTADQESLEKR(88) IAEEWAPASR(60) LGKPEIFPASTDAR(92) AYASYVDGGISAVPR(62) AYASYVDGGISAVPRDEL(100) |
| 40 | MDP0000301987 | Ketol-acid reductoisomerase, chloroplastic | 1.64 | 0.0250 | 684 | 18 | 7 | 66.606/8.08 | EKINLAGHNEYIVR(119) INLAGHNEYIVR(90) DLFHLLPDAFK(87) QIGVIGWGSQGPAQAQNLR(160) EINGAGINSSFAVHQDVDGR(153) NTVECITGIVSR(47) GVSFMVDNCSTTAR(93) |
| 41 | MDP0000300217 | Phospholipase D alpha | 0.40 | 0.0398 | 212 | 10 | 6 | 94.522/5.83 | ESNPIGASLIGR(22) FPGVPFTFFSQR(50) LEGPIAWDVLFNFEQR(42) SIDGGAAFGFPDTPEDAAR(114) SIQDAYIHAIR(29) KPGEYEPSETPEADSDYQR(98) |
| 42 | MDP0000096349 | Glutathione S-transferase | 1.82 | 0.0056 | 269 | 42 | 8 | 24.028/6.17 | VHGNVISTAAMR(58) VFATLYEKDIEFELVPIDMR(53) DIEFELVPIDMR(69) DIEFELVPIDMR(44) EPFISLNPFGQVPAFEDGDLK(76) AITQYIAHEYADK(98) MTTDAAVVEENEAK(25) LAVVLDVYETR(66) |
| 43 | MDP0000269612 | Cinnamoyl-CoA reductase | 0.23 | 0.0263 | 438 | 25 | 7 | 39.339/5.91 | HLEALVEGAESR(87) VKGEDCWTDIDYCK(95) GEDCWTDIDYCK(73) GLDVVVVNPGTVMGPVISPR(119) ILEGCTETYEDFFMGSVHFK(107) DVALAHILVYENK(118) HLCLEAISR(59) |
| 44 | MDP0000868045 | Abscisic acid response protein | 2.43 | 0.0098 | 235 | 17 | 3 | 19.860/8.61 | SHLHNQHGAQPTDAYGNPIQR (165) KEHHGVTGALHR (55)  EHHGVTGALHR (79) |
| 45 | MDP0000868045 | Abscisic acid response protein | 2.91 | 0.0011 | 241 | 17 | 2 | 19.860/8.61 | SHLHNQHGAQPTDAYGNPIQR (182)  EHHGVTGALHR (92) |
| 46 | MDP0000148984 | Methylthioribose kinase | 1.79 | 0.0025 | 195 | 18 | 4 | 32.873/5.33 | AFSEFRPLDEK(48) EHGSLSPDHVPEVYHFDR(103) AVAEFCGNVELCR(82) LTEQVVFSDPYK(68) |
| 47 | MDP0000868045 | Abscisic acid response protein | 2.33 | 0.0406 | 285 | 17 | 3 | 19.860/8.61 | SHLHNQHGAQPTDAYGNPIQR (190) KEHHGVTGALHR (68)  EHHGVTGALHR (99) |
| 48 | MDP0000868045 | Abscisic acid response protein | 2.21 | 0.0286 | 258 | 26 | 3 | 19.860/8.61 | SHLHNQHGAQPTDAYGNPIQR (165) EHHGVTGALHR (92)  SGSSSSSSSEDDGLGGR (64) |
| 49 | MDP0000322880 | Nucleoside diphosphate kinase | 2.10 | 0.0463 | 290 | 35 | 6 | 18.663/7.96 | GLVGDIISR(44) FINVDRPFAEK(69) KIIGATNPAESAPGTIR(111) IIGATNPAESAPGTIR(121) GDYAIEIGR(47) NIIHGSDSAEGAR(89) |
| 50 | MDP0000376563 | Protein phosphatase 2c-like protein | 2.33 | 0.0180 | 177 | 18 | 6 | 38.261/7.79 | NILKEPDFWTEPK(57) EPDFWTEPK(45) QLSVDHEPSTEREDIENR(42) GGFVSNFPGDVPR(107) VDGQLAVAR(48) SSDDISCIVVR(39) |
| 51 | MDP0000014145 | Proteasome subunit beta type-7 | 0.24 | 0.0216 | 158 | 22 | 4 | 29.403/7.14 | DGVILGADTR(34) ATEGPIVCDKNCEK(72) LVTEAICSGIFNDLGSGSNVDVCVITK(101) NHLVPTPR(41) |
| 52 | MDP0000273688 | Fructose-bisphosphate aldolase | 1.70 | 0.0129 | 426 | 16 | 8 | 67.338/8.59 | RLDSIGLDNTEVNR(71) LDSIGLDNTEVNR(58) FVDVLCDQK(46) GLVPLPGSNNESWCQGLDGLASR(149) TVVSIPCGPSALAVK(49) YAAISQDNGLVPIVEPEILLDGDHPIER(194) AWQGRPENVEAAQK(77) AWQGRPENVEAAQK(30) |
| 53 | MDP0000165865 | Uridine 5'-monophosphate synthase | 0.30 | 0.0448 | 192 | 24 | 3 | 20.535/6.73 | ASLPSTESLILQLHDISAVK(125) SGISSPIYIDLR(69) EQGGRENLEENGIR(70) |
| 54 | MDP0000261821 | Monodehydroascorbate reductase | 1.61 | 0.0477 | 587 | 27 | 9 | 47.084/6.51 | YVILGGGVSAGYAAR(137) EAVAPYERPALSK(78) AYLLPESPAR(30) AYLFPESPAR(40) LPGFHVCVGSGGER(77) TLVSGTGESFKYETLVIATGSTVIR(240) AVIVGGGYIGLELGAALR(125) MVYPEPWCMPR(15) TVEEYDYLPFFYSR(99) |
| 55 | MDP0000179036 | Enolase | 1.56 | 0.0354 | 119 | 9 | 3 | 47.936/5.77 | AAVPSGASTGVYEALELR(56) VNQIGSVTESIEAVR(96) FRVPVEPY(23) |
| 56 | MDP0000166687 | 14-3-3 protein | 1.78 | 0.0044 | 372 | 24 | 9 | 46.632/5.75 | ASAVPENLSR (45)  EQYVYLAK (46)  LAEQAERYEEMVSFMEK (61) LVVGSTAAGTELTVEER (140)  IISSIEQKEEGR (90)  YLAEFKNGDER (42)  AAQDIALADLAPTHPIR (137)  DSTLIMQLLR (60) DSTLIMQLLR (19) |
| 57 | MDP0000699845 | Polyphenol oxidase | 1.89 | 0.0423 | 127 | 8 | 4 | 63.227/7.57 | IRPPAHAVDQAYR (55)  YYLYFFEK (47)  LFFGNPYR (41)  NLGYVYQDVDIPWLSSKPTPR (79) |

^a^ Statistical analysis was performed on all the protein spots that exhibited 1.5-fold cutoff (ratio above 1.5 or below 1/1.5) with *P*-value< 0.05 in ALA-treated apple skin compred to control. The corresponding spots were extracted from duplicated 2-DE gels and identified by MS.
