# Supplementary material for: Proteomics and SSH Analyses of ALA-Promoted Fruit Coloration and Evidence for the Involvement of a MADS-Box Gene, MdMADS1
Source: Front Plant Sci. 2016 Nov 7;7:1615. doi: 10.3389/fpls.2016.01615 (PMC5098116; doi:10.3389/fpls.2016.01615)
Supplement: Supplementary file 3 [file Table3.DOC]

**Table S3 Statistically significant different expressed proteins obtained by gel-free analysis in ALA-treated apple skin**

| Accession No. | Annotation | Peptides | Frames | Hits | Ratio | *P*-value |
| --- | --- | --- | --- | --- | --- | --- |
| MDP0000325949 | 14-3-3 protein family | 4 | 5 | 32 | 6.68 | 0.0000 |
| MDP0000442105 | LYR family of Fe/S cluster biogenesis protein | 2 | 2 | 5 | 5.69 | 0.0000 |
| MDP0000279955 | Uncharacterized protein | 2 | 2 | 13 | 4.60 | 0.0000 |
| MDP0000450991 | Lipoxygenase | 3 | 3 | 18 | 4.54 | 0.0000 |
| MDP0000452572 | Universal stress protein (USP) family protein | 7 | 7 | 53 | 3.58 | 0.0000 |
| MDP0000147916 | Serine acetyltransferase | 2 | 2 | 8 | 3.45 | 0.0000 |
| MDP0000519575 | Peroxiredoxin | 4 | 4 | 16 | 3.35 | 0.0000 |
| MDP0000527995 | Glyceraldehyde-3-phosphate dehydrogenase | 2 | 2 | 10 | 2.49 | 0.0000 |
| MDP0000280265 | Acidic endochitinase | 2 | 2 | 11 | 2.27 | 0.0000 |
| MDP0000153379 | L-arabinokinase | 6 | 6 | 23 | 2.26 | 0.0000 |
| MDP0000284588 | Glutamate decarboxylase | 3 | 3 | 23 | 2.16 | 0.0000 |
| MDP0000796883 | Adenine nucleotide translocator | 5 | 5 | 21 | 2.04 | 0.0000 |
| MDP0000269483 | Xyloglucan endotransglucosylase/hydrolase protein 6 | 3 | 3 | 20 | 2.01 | 0.0000 |
| MDP0000543784 | Uncharacterized protein | 2 | 2 | 10 | 1.96 | 0.0000 |
| MDP0000183725 | Pyruvate kinase | 3 | 4 | 13 | 1.85 | 0.0001 |
| MDP0000597996 | Ribulose-1-5-bisphosphate carboxylase | 3 | 3 | 19 | 1.82 | 0.0158 |
| MDP0000273688 | Fructose-bisphosphate aldolase 3 | 6 | 7 | 36 | 1.82 | 0.0000 |
| MDP0000298613 | Ubiquinol-cytochrome c reductase complex 14 kDa protein | 2 | 2 | 2 | 1.80 | 0.0010 |
| MDP0000195885 | 1-aminocyclopropane-1-carboxylate oxidase 1 | 8 | 12 | 81 | 1.80 | 0.0004 |
| MDP0000609966 | Polyphenol oxidase | 2 | 2 | 12 | 1.79 | 0.0164 |
| MDP0000320612 | Peroxiredoxin | 2 | 2 | 6 | 1.71 | 0.0004 |
| MDP0000199034 | L-ascorbate peroxidase | 3 | 4 | 14 | 1.71 | 0.0070 |
| MDP0000615956 | 4-coumarate-CoA ligase | 4 | 4 | 30 | 1.70 | 0.0000 |
| MDP0000321341 | Pyrophosphate-D-fructose 6-phosphate 1-phosphotransferase | 5 | 6 | 29 | 1.70 | 0.0000 |
| MDP0000217005 | Transketolase | 2 | 2 | 5 | 1.67 | 0.0000 |
| MDP0000616695 | 60S ribosomal protein L11 isoform X1 | 2 | 2 | 11 | 1.66 | 0.0009 |
| MDP0000219737 | Ethylene receptor 2 | 2 | 1 | 2 | 1.61 | 0.0000 |
| MDP0000239328 | Methylmalonate-semialdehyde dehydrogenase | 2 | 2 | 2 | 1.60 | 0.0000 |
| MDP0000322880 | Nucleoside diphosphate kinase | 2 | 2 | 20 | 1.58 | 0.0000 |
| MDP0000246775 | Thaumatin-like protein 1a | 3 | 4 | 28 | 1.58 | 0.0098 |
| MDP0000913598 | Glutathione peroxidase | 2 | 2 | 5 | 1.54 | 0.0000 |
| MDP0000052862 | UDP-glucose: anthocyanidin 3-O-glucosyltransferase | 2 | 2 | 16 | 1.53 | 0.0179 |
| MDP0000268037 | NADP-dependent malic enzyme | 9 | 14 | 90 | 1.51 | 0.0000 |
| MDP0000249227 | Soluble inorganic pyrophosphatase | 2 | 2 | 3 | 1.51 | 0.0000 |
| MDP0000770493 | Dehydrin-like protein | 7 | 15 | 62 | 0.66 | 0.0012 |
| MDP0000250284 | Uncharacterized protein | 2 | 2 | 3 | 0.66 | 0.0001 |
| MDP0000251031 | Uncharacterized protein | 2 | 2 | 4 | 0.66 | 0.0337 |
| MDP0000096349 | Glutathione-S-transferase | 5 | 5 | 19 | 0.65 | 0.0000 |
| MDP0000288293 | Major allergen Pru ar 1 | 5 | 6 | 32 | 0.65 | 0.0002 |
| MDP0000121897 | Adenine phosphoribosyltransferase 1 | 2 | 2 | 15 | 0.64 | 0.0370 |
| MDP0000297664 | Putative mitochondrial 2-oxoglutarate/malate carrier protein | 2 | 2 | 7 | 0.64 | 0.0001 |
| MDP0000940078 | Plant lipid transfer protein | 2 | 3 | 11 | 0.58 | 0.0458 |
| MDP0000277802 | MLP-like protein 329 | 4 | 4 | 19 | 0.55 | 0.0000 |
| MDP0000253074 | Abscisic acid stress ripening protein homolog | 8 | 18 | 110 | 0.53 | 0.0000 |
| MDP0000256937 | 40S ribosomal protein S3-3-like | 2 | 2 | 12 | 0.51 | 0.0000 |
| MDP0000147610 | F-type ATPases | 2 | 2 | 13 | 0.50 | 0.0000 |
| MDP0000668552 | Glutamine amidotransferase-like Class I superfamily protein | 2 | 2 | 6 | 0.19 | 0.0000 |

* Proteins satisfying all the following criteria may be chosen as differentially expressed: Matched peptides≥2, 1.5-fold cutoff (ratio above 1.5 or below 1/1.5) and *P*-value < 0.05.
