# Supplementary material for: Proteomics and SSH Analyses of ALA-Promoted Fruit Coloration and Evidence for the Involvement of a MADS-Box Gene, MdMADS1
Source: Front Plant Sci. 2016 Nov 7;7:1615. doi: 10.3389/fpls.2016.01615 (PMC5098116; doi:10.3389/fpls.2016.01615)
Supplement: Supplementary file 4 [file Table4.DOC]

**Table S4** Twelve proteins commonly identified by the gel-based and gel-free proteomics techniques.

| Accession No. | Annotation |
| --- | --- |
| MDP0000249227 | Soluble inorganic pyrophosphatase |
| MDP0000147610 | F-type ATPases |
| MDP0000273688 | Fructose-bisphosphate aldolase 3 |
| MDP0000195885 | 1-aminocyclopropane-1-carboxylate oxidase 1 |
| MDP0000121897 | Adenine phosphoribosyltransferase 1 |
| MDP0000322880 | Nucleoside diphosphate kinase 1 |
| MDP0000609966 | Polyphenol oxidase |
| MDP0000052862 | UDP-glucose: anthocyanidin 3-O-glucosyltransferase |
| MDP0000096349 | Glutathione-S-transferase |
| MDP0000277802 | MLP-like protein 329 |
| MDP0000199034 | L-ascorbate peroxidase |
| MDP0000246775 | Thaumatin-like protein 1a |
