# Supplementary material for: Proteomics and SSH Analyses of ALA-Promoted Fruit Coloration and Evidence for the Involvement of a MADS-Box Gene, MdMADS1
Source: Front Plant Sci. 2016 Nov 7;7:1615. doi: 10.3389/fpls.2016.01615 (PMC5098116; doi:10.3389/fpls.2016.01615)
Supplement: Supplementary file 5 [file Table5.DOC]

**Table S5 ALA-induced differentially expressed genes obtained by SSH analysis** in apple skin

| Unigene No. | Accession No. | Length (bp) | Annotation |
| --- | --- | --- | --- |
| UN001 | MDP0000864747 | 510 | Major allergen mal d 1 |
| UN002 | MDP0000155675 | 436 | Rieske iron-sulfur protein tic55 |
| UN003 | MDP0000170439 | 310 | Uncharacterized protein |
| UN004 | MDP0000668552 | 503 | Class I glutamine amidotransferase-like superfamily protein |
| UN005 | MDP0000251991 | 686 | Lipid transport superfamily protein |
| UN006 | MDP0000159070 | 400 | Alpha-1,2-fucosidase |
| UN007 | MDP0000170162 | 452 | UDP-glucose: anthocyanidin 5,3-O-glucosyltransferase |
| UN008 | MDP0000292256 | 362 | Mitochondrial pyruvate carrier 2 |
| UN009 | MDP0000792008 | 520 | Protease Do-like 1, chloroplastic |
| UN010 | MDP0000169491 | 503 | Protease Do-like 10, mitochondrial |
| UN011 | MDP0000213188 | 232 | Uncharacterized protein |
| UN012 | MDP0000364214 | 404 | Uncharacterized protein |
| UN013 | MDP0000184228 | 968 | Pectinesterase-like |
| UN014 | MDP0000236092 | 120 | COBRA-like protein 10 |
| UN015 | MDP0000906581 | 385 | Protein serine/threonine kinase |
| UN016 | MDP0000281005 | 495 | Pyruvate phosphate dikinase |
| UN017 | MDP0000316126 | 521 | Phytosulfokine family protein |
| UN018 | MDP0000142957 | 364 | Probable aquaporin PIP2-8 |
| UN019 | MDP0000128787 | 801 | Photosystem I reaction centre subunit IV |
| UN020 | MDP0000127542 | 489 | β-galactosidase (BGAL101) |
| UN021 | MDP0000844309 | 446 | Transmembrane 9 superfamily member 2 |
| UN022 | MDP0000770493 | 313 | Dehydrin family |
| UN023 | MDP0000253903 | 534 | Uncharacterized protein |
| UN024 | MDP0000176633 | 318 | Polyadenylate-binding protein-interacting protein 11-like |
| UN025 | MDP0000319681 | 365 | PUA domain-containing protein |
| UN026 | MDP0000245842 | 480 | Ubiquitin-associated/translation elongation factor EF1B |
| UN027 | MDP0000940313 | 599 | Acidic endochitinase |
| UN028 | MDP0000294774 | 302 | Proteasome subunit beta type-2-A |
| UN029 | MDP0000315400 | 391 | Uncharacterized Protein |
| UN030 | MDP0000270142 | 228 | Uncharacterized protein |
| UN031 | MDP0000140404 | 323 | methylcrotonoyl-CoA carboxylase |
| UN032 | MDP0000139843 | 189 | Uncharacterized protein |
| UN033 | MDP0000332599 | 266 | dihydrolipoyllysine-residue succinyltransferase component of 2-oxoglutarate dehydrogenase complex mitochondrial-like |
| UN034 | MDP0000678574 | 288 | Quinone oxidoreductase |
| UN035 | MDP0000224466 | 449 | WUSCHEL-related homeobox 8-like |
| UN036 | MDP0000240055 | 398 | Raffinose synthase 5 |
| UN037 | MDP0000452572 | 378 | Universal stress protein A-like protein |
| UN038 | MDP0000366022 | 360 | MADS1 |
| UN039 | MDP0000942516 | 749 | Major allergen mal d 1 |
| UN040 | MDP0000295542 | 364 | Major allergen Mal d 1 |
| UN041 | MDP0000183572 | 579 | Vesicle-associated protein 2-1 |
| UN042 | MDP0000196182 | 291 | NADH dehydrogenase [ubiquinone] iron-sulfur protein 6, mitochondrial |
| UN043 | MDP0000199152 | 493 | α-farnesene synthase |
| UN044 | MDP0000171041 | 453 | S-adenosylmethionine decarboxylase |
| UN045 | MDP0000595966 | 271 | ClpP family protein |
| UN046 | MDP0000250936 | 382 | Glycosyl hydrolase 38 |
| UN047 | MDP0000319369 | 314 | Glycosyltransferase |
| UN048 | MDP0000192559 | 377 | GDT1-like protein 3 |
| UN049 | MDP0000624481 | 596 | SAD1/UNC-84 domain protein 1 |
| UN050 | MDP0000161521 | 331 | Auxin-responsive family protein |
| UN051 | MDP0000237977 | 1090 | Uncharacterized protein |
| UN052 | MDP0000904060 | 376 | WD40 repeat-like superfamily protein//USP1-associated factor 1 |
| UN053 | MDP0000928106 | 585 | Uncharacterized protein |
| UN054 | MDP0000231245 | 648 | Probable indole-3-acetic acid-amido synthetase GH3.6 |
| UN055 | MDP0000570395 | 155 | Glucan endo-1,3-beta-glucosidase |
| UN056 | MDP0000148978 | 632 | Phytoene dehydrogenase |
| UN057 | MDP0000262512 | 701 | lipases;hydrolases, acting on ester bonds |
| UN058 | MDP0000237591 | 169 | SBP (S-ribonuclease binding protein) family protein |
| UN059 | MDP0000501504 | 139 | Alcohol dehydrogenase |
| UN060 | MDP0000258968 | 396 | Probable protein phosphatase 2C 60 |
| UN061 | MDP0000329597 | 370 | DNA repair ATPase-related family protein |
| UN062 | MDP0000789873 | 274 | Glycoside hydrolase |
| UN063 | MDP0000669032 | 841 | Uncharacterized protein |
| UN064 | MDP0000275042 | 517 | High mobility group B protein 3 |
| UN065 | MDP0000889408 | 316 | ARF guanyl-nucleotide exchange factor |
| UN066 | MDP0000872932 | 477 | Nucleolar GTP-binding protein 2-like |
| UN067 | MDP0000337709 | 450 | Armadillo-type fold domain |
| UN068 | MDP0000253390 | 698 | phosphoenolpyruvate carboxylase-related kinase 2 |
| UN069 | MDP0000153123 | 433 | Metallothionein-like protein |
| UN070 | MDP0000621545 | 310 | Acetolactate synthase |
| UN071 | MDP0000263908 | 627 | 40S ribosomal protein S27 |
| UN072 | MDP0000188613 | 816 | Desiccation-related protein PCC13-62 |
| UN073 | MDP0000128287 | 403 | Zinc finger protein ZPR1 homolog |
| UN074 | MDP0000228673 | 553 | Dehydration-responsive protein RD22-like |
| UN075 | MDP0000253152 | 633 | Predicted nucleic acid binding protein |
| UN076 | MDP0000164592 | 640 | NADH dehydrogenase [ubiquinone] iron-sulfur protein 8 |
| UN077 | MDP0000103627 | 161 | Major allergen Mal d 1 |
| UN078 | MDP0000281971 | 639 | Photosystem I reaction centre subunit IV |
| UN079 | MDP0000130449 | 259 | cytochrome P450 monooxygenase |
| UN080 | MDP0000874088 | 590 | Somatic embryogenesis receptor kinase 2 |
| UN081 | MDP0000272753 | 365 | Uncharacterized protein |
| UN082 | MDP0000674266 | 355 | 40s ribosomal protein s25 |
| UN083 | MDP0000250621 | 473 | IST1 homolog |
| UN084 | MDP0000241086 | 558 | Surfeit locus protein isoform |
| UN085 | MDP0000222113 | 415 | Ubiquitin-protein ligase 10/12 |
| UN086 | MDP0000647505 | 344 | photosystem II core complex proteins psbY, chloroplastic-like |
| UN087 | MDP0000240250 | 358 | lipid phosphate phosphohydrolase |
| UN088 | MDP0000925483 | 627 | Transaldolase |
| UN089 | MDP0000325949 | 167 | 14-3-3 protein family |
| UN090 | MDP0000255495 | 407 | Uncharacterized protein |
| UN091 | MDP0000292565 | 643 | Uncharacterized Protein |
| UN092 | MDP0000267248 | 549 | 6-phosphofructokinase |
| UN093 | MDP0000253074 | 236 | Abscisic acid stress ripening protein homolog |
| UN094 | MDP0000165546 | 374 | ClpS family protein |
| UN095 | MDP0000195885 | 438 | 1-aminocyclopropane-1-carboxylate oxidase 1 |
| UN096 | MDP0000198736 | 329 | Farnesyl pyrophosphate synthase |
| UN097 | MDP0000291707 | 402 | NAD(P)-binding Rossmann-fold superfamily protein |
| UN098 | MDP0000456397 | 278 | Cullin 4 |
| UN099 | MDP0000156410 | 253 | Uncharacterized protein |
| UN100 | MDP0000145603 | 513 | Transcription factor TFIIE, alpha subunit |
| UN101 | MDP0000305778 | 667 | Acyl-CoA-binding protein 6 |
| UN102 | MDP0000215630 | 344 | BAG family molecular chaperone regulator 4-like |
| UN103 | MDP0000249772 | 498 | S-adenosyl-L-methionine:carboxyl methyltransferase protein |
| UN104 | MDP0000233778 | 357 | Thylakoid lumen 15.0 kDa protein |
