# Supplementary material for: Proteomics and SSH Analyses of ALA-Promoted Fruit Coloration and Evidence for the Involvement of a MADS-Box Gene, MdMADS1
Source: Front Plant Sci. 2016 Nov 7;7:1615. doi: 10.3389/fpls.2016.01615 (PMC5098116; doi:10.3389/fpls.2016.01615)
Supplement: Supplementary file 6 [file Image1.PDF]

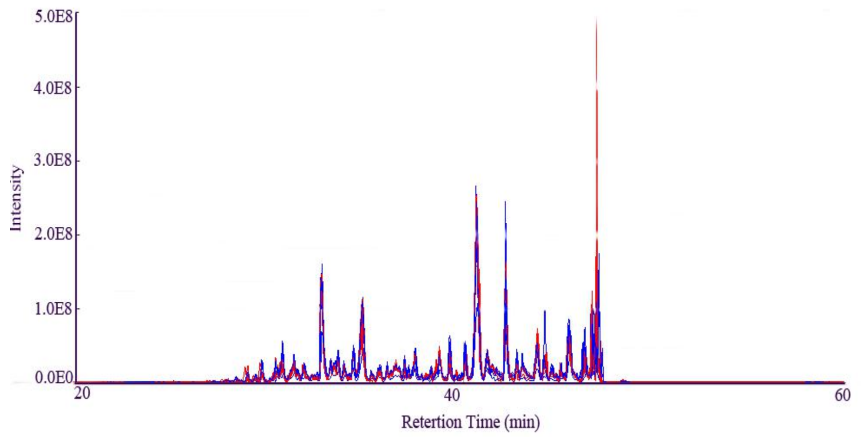

Figure S1. For label-free relative quantitation, chromatographic peaks of control (blue) and ALA-treated samples (red) were aligned by retention time (± 2.5 min) and mass (±0.02 unit) in SEIVE analysis.
